# Grounded reconfigurable metamaterials with customized mapping-invariant behavior

**DOI:** 10.1038/s41467-026-73240-8

**Published:** 2026-05-19

**Authors:** Yu Huang, Yuxuan Tang, Zhengyu Li, Michael R. Haberman, Yangyang Chen

**Affiliations:** 1 Department of Mechanical and Aerospace Engineering, The Hong Kong University of Science and Technology, Kowloon, Hong Kong; 2 Walker Department of Mechanical Engineering, University of Texas at Austin, Austin, TX USA

**Keywords:** Mechanical engineering, Mechanical properties

## Abstract

Mechanical behavior of synthetic materials depends on their microstructure and geometric configurations. This dependency leads to unintended performance when the material remaps its microstructures for shape reconfigurations, such as diminished rigidity in unfolded aerospace morphing structures and reduced sensitivity in twisted soft sensors. Breaking this dependency through material design to improve overall performance has been a long-standing challenge. This work develops a transformation method to design a class of grounded metamaterials that decouples mechanical behavior from microstructure and shape reconfigurations. We fabricate these metamaterials and experimentally demonstrate both configuration-mapping-invariant displacement behavior and unconventional displacement control functions that have not previously been observed. We identify two physical principles that underpin the useful, but counterintuitive behavior: (i) Mapping-invariant displacement fields are the result of body torques that automatically balance non-concurrent internal forces from microstructure reconfigurations; (ii) Tailored displacement control functions are determined by Willis springs pinned to the ground. As a result, the grounded metamaterials are shown to enable the design of highly reconfigurable material systems that demonstrate tailored deformation behavior regardless of their microscopic and geometric configurations.

## Abstract (web summary)

The work presents a method to design grounded metamaterials whose displacement response remains unchanged under microstructure and shape reconfiguration, demonstrated experimentally using structures with tailored, mapping-invariant deformation behaviours.

## Introduction

Mechanical behavior of synthetic materials is dictated by their microstructure and geometric configurations, a principle that drives research and development of composites and mechanical metamaterials^1–6^. Typically, composites and metamaterials are designed with optimal microstructure and geometric configurations for particular functions, i.e., load bearing^7–10^, energy absorption^11–14^, wave control^15–18^, adaptive locomotion^19,20^, and shape morphing^21,22^. However, mechanical metamaterials often fail to preserve their intended performance after microstructure reconfigurations to achieve the desired shape transformations. For example, shape reconfigurations in multi-stable metamaterials can cause unintended snapping or incomplete closure^23^, inflatable space structures, when deployed, face compromised stability^24^, and twisted soft sensors or actuators, exhibit weak electromechanical coupling^25^. These examples highlight the long-standing trade-off between geometric reconfigurability and performance of intended functions. Designing mechanical metamaterials to decouple performance from microstructure and shape reconfigurations remains elusive.

The present work develops a transformation method to design grounded metamaterials (GMMs) with desired displacement responses that are invariant to microstructure and shape reconfiguration. Transformation methods have been developed to calculate spatially varying material parameters for cloak and lens designs in optics^26–28^, acoustics^29–31^, and elasticity^32–34^, where form-invariance of governing equations ensures that small amplitude waves can propagate through materials of any shape without scattering. In this work, we leverage coordinate transformation to describe geometric mapping of microstructures in discrete metamaterials and build a transformation rule for material parameters to decouple displacement responses from reconfigurations. Unlike optics and acoustics, transformation elasticity requires that transformed solid materials be polar and/or exhibit Willis coupling^35–41^. In what follows, we introduce a GMM that displays polarity and Willis coupling, which can automatically satisfy the material parameter requirements from the transformation method. We then construct the GMMs and demonstrate the arbitrary, yet mapping-invariant, displacement fields in experiments. This work demonstrates two physical principles that govern the behavior of the GMMs derived from the transformation: (i) mapping-invariant displacement fields in GMMs are the result of the body torques that balance nonconcurrent internal forces from reconfigurations; (ii) tailored displacement control functions are determined by Willis springs pinned to the ground. As a result, we demonstrate that GMMs enable the creation of highly adjustable material systems invariant to their configurations.

## Results

### Design GMMs based on the transformation method

**Fig. 1:**
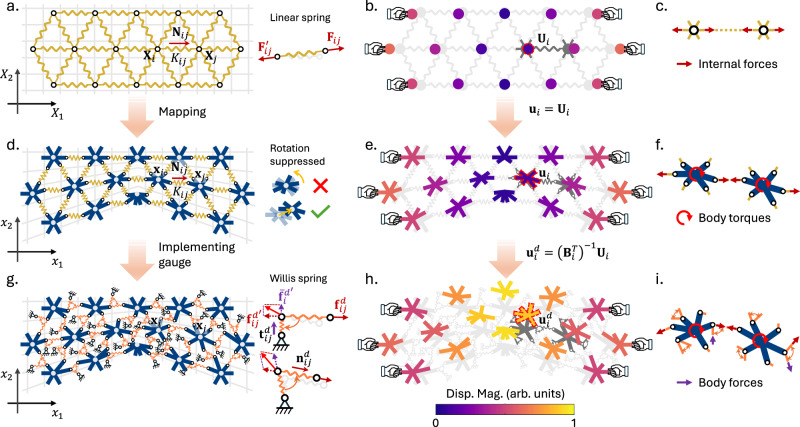
Design grounded metamaterials (GMMs) based on the transformation method. **a** A free-standing lattice composed of pin-connected linear springs. **b** The free-standing lattice is deformed by uniaxial tension. **c** The concurrent internal forces on two adjacent nodes in (**b**) (shown in dark gray). **d** The GMM derived from coordinate transformation, where rotations of reconfigurable masses are suppressed while translational motion is free. **e** The GMM is deformed by the same uniaxial tension as that in (**b**), where the displacement field remains unchanged. **f** The nonconcurrent internal forces on two adjacent masses in (**e**) (shown in dark gray), and reaction body torques are exerted on the masses. **g** The GMM composed of Willis springs and rotation-suppressed reconfigurable masses derived by the transformation method, where a customized displacement gauge is implemented. **h** The deformation of the GMM (shown in (**g**)) by the same uniaxial tension as that in (**b**). Customized tension-induced buckling is achieved. **i** The internal forces, body torques, and body forces on two adjacent masses in (**h**) (shown in dark gray).

We first consider a free-standing lattice, composed of pin-connected linear springs, before the transformation (see Fig. 1a). The linear spring between nodes **X**_*i*_ and **X**_*j*_ (along **N**_*ij*_ direction) hosts an internal force **F**_*ij*_ and has a spring constant *K*_*ij*_. When the free-standing lattice is subjected to external loads, the lattice deforms with displacement **U**_*i*_ at the node **X**_*i*_ (see the deformation under uniaxial tension in Fig. 1b). Clearly, the net internal force on each node is zero, and no torques appear. Figure 1c shows the concurrent internal forces when the lattice is under uniaxial tension.

To maintain the same displacement response after microstructure and shape reconfigurations, our strategy is to describe reconfigured node positions by the geometric mapping from **X**_*i*_ to **x**_*i*_ and perform coordinate transformation so that the elastic energy of each of the linear springs remains unchanged when the lattice of the two configurations deforms under the same external constraints. That is ${e}_{ij}=\frac{1}{2}{k}_{ij}{[({{{{\bf{u}}}}}_{j}-{{{{\bf{u}}}}}_{i})\cdot {{{{\bf{n}}}}}_{ij}]}^{2}={E}_{ij}=\frac{1}{2}{K}_{ij}{[({{{{\bf{U}}}}}_{j}-{{{{\bf{U}}}}}_{i})\cdot {{{{\bf{N}}}}}_{ij}]}^{2}$, where **u**_*i*_, *k*_*ij*_, and **n**_*ij*_ are the displacement, spring stiffness, and direction of the transformed lattice (see Fig. 1d). Enforcing **u**_*i*_ = **U**_*i*_ leads to *k*_*ij*_ = *K*_*ij*_ and **n**_*ij*_ = **N**_*ij*_. Consequently, to achieve the same displacement field, the spring constant and direction would have to remain unchanged before and after the transformation associated with lattice reconfiguration. To comply with this requirement, we employ the reconfigurable mass at each of the nodes (the starburst structure with six stacked arms connected by a screw shown in Fig. 1d) to construct the lattice. This more general lattice structure allows substantial microstructure and geometric reconfiguration while maintaining the same spring constant and direction by loosening the screw, adjusting the shape of the starburst structure, and tightening the screw. Note that we consider reconfigurable masses of different shapes as rigid bodies in the lattice. Further, since the reconfigurable masses function as point masses, as those in the free-standing lattice, the rotational degrees of freedom of the reconfigurable masses must be suppressed while the translational motion is free (see Fig. 1d). We realize this requirement using grounded mechanisms (see the detailed design in the next section). This way, the lattice preserves the displacement field, invariant to microstructure and shape reconfiguration (see the illustrative example of uniaxial tension in Fig. 1e). It is worth mentioning that the internal spring forces **F**_*ij*_ and **f**_*ij*_ also remain the same in configurations **X**_*i*_ and **x**_*i*_. As a result, the net internal force on the reconfigurable mass is still zero, but the net torque produced by the internal forces is nonzero, since the internal forces are no longer concurrent (see the simple example in Fig. 1f). Thus, a reaction body torque is exerted on the reconfigurable mass due to its suppressed rotation. The body torques in the grounded lattice metamaterial are the key to generating the mapping-invariant displacement field.

Next, we exploit the possibility of achieving a customized displacement field ${{{{\bf{u}}}}}_{i}^{d}\ne {{{{\bf{u}}}}}_{i}$ in the configuration **x**_*i*_, which, at the same time, is also mapping-invariant (see Fig. 1g). To achieve ${{{{\bf{u}}}}}_{i}^{d}$, we consider the GMM shown in Fig. 1d as the reference and define a displacement gauge **B**_*i*_ as ${{{{\bf{u}}}}}_{i}^{d}={({{{{\bf{B}}}}}_{i}^{T})}^{-1}{{{{\bf{u}}}}}_{i}$. Implementing the displacement gauge into the transformation, the elastic energy of each of the linear springs still remains unchanged, ${e}_{ij}^{d}={e}_{ij}$. Substituting the definition of the displacement gauge into *e*_*ij*_, the elastic energy ${e}_{ij}^{d}=\frac{1}{2}{k}_{ij}{[({{{{\bf{u}}}}}_{j}^{d}-{{{{\bf{u}}}}}_{i}^{d})\cdot {{{{\bf{B}}}}}_{j}{{{{\bf{n}}}}}_{ij}-{{{{\bf{u}}}}}_{i}^{d}\cdot ({{{{\bf{B}}}}}_{i}-{{{{\bf{B}}}}}_{j}){{{{\bf{n}}}}}_{ij}]}^{2}$. To satisfy the form of the elastic energy ${e}_{ij}^{d}$, the linear spring after the transformation should contain two coupled springs (see Fig. 1g). One is an internal spring linking masses, and the other is a grounded spring connected to a reference ground. The internal and grounded spring forces read

1 $${{{{\bf{f}}}}}_{ij}^{d}=\left[{k}_{ij}^{d}({{{{\bf{u}}}}}_{j}^{d}-{{{{\bf{u}}}}}_{i}^{d})\cdot {{{{\bf{n}}}}}_{ij}^{d}-{\widehat{k}}_{ij}^{d}{{{{\bf{u}}}}}_{i}^{d}\cdot {{{{\bf{t}}}}}_{ij}^{d}\right] \, {{{{\bf{n}}}}}_{ij}^{d},$$

2 $${\overline{{{{\bf{f}}}}}}_{i}^{d}=\left[{\widehat{k}}_{ij}^{d}({{{{\bf{u}}}}}_{j}^{d}-{{{{\bf{u}}}}}_{i}^{d})\cdot {{{{\bf{n}}}}}_{ij}^{d}-{\widetilde{k}}_{ij}^{d}{{{{\bf{u}}}}}_{i}^{d}\cdot {{{{\bf{t}}}}}_{ij}^{d}\right] \, {{{{\bf{t}}}}}_{ij}^{d},$$

where ${k}_{ij}^{d}={k}_{ij}{| {{{{\bf{B}}}}}_{j}{{{{\bf{n}}}}}_{ij}| }^{2}$, ${\widehat{k}}_{ij}^{d}={k}_{ij}| {{{{\bf{B}}}}}_{j}{{{{\bf{n}}}}}_{ij}| | ({{{{\bf{B}}}}}_{i}-{{{{\bf{B}}}}}_{j}){{{{\bf{n}}}}}_{ij}|$, ${\widetilde{k}}_{ij}^{d}={k}_{ij}{| ({{{{\bf{B}}}}}_{i}-{{{{\bf{B}}}}}_{j}){{{{\bf{n}}}}}_{ij}| }^{2}$, ${{{{\bf{n}}}}}_{ij}^{d}={{{{\bf{B}}}}}_{j}{{{{\bf{n}}}}}_{ij}/| {{{{\bf{B}}}}}_{j}{{{{\bf{n}}}}}_{ij}|$, and ${{{{\bf{t}}}}}_{ij}^{d}=({{{{\bf{B}}}}}_{i}-{{{{\bf{B}}}}}_{j}){{{{\bf{n}}}}}_{ij}/| ({{{{\bf{B}}}}}_{i}-{{{{\bf{B}}}}}_{j}){{{{\bf{n}}}}}_{ij}|$. In the equations, ${k}_{ij}^{d}$ and ${\widetilde{k}}_{ij}^{d}$ denote the spring constants of the internal and grounded springs, respectively, and ${{{{\bf{n}}}}}_{ij}^{d}$ and ${{{{\bf{t}}}}}_{ij}^{d}$ are their directions. Clearly, a change in length of the grounded spring causes a force in the internal spring and vice versa, a behavior characterized by a Willis coupling coefficient ${\hat{k}}_{ij}^{d}$ (see Fig. 1g). We call the coupled springs a Willis spring. Assembling Willis springs in the transformed lattice following Eqs. 1 and 2, the customized displacement field ${{{{\bf{u}}}}}_{i}^{d}$ can be achieved automatically under the same external constraints (see Fig. 1h for the desired tension-induced buckling). Note that the rotation-suppressed reconfigurable masses before the transformation are preserved in the grounded Willis lattice. As shown in Fig. 1i, the grounded connection in the Willis spring provides a tailored body force applied on the reconfigurable mass that flexibly manipulates the displacement field of the Willis lattice. It is worth mentioning that the displacement field of the Willis lattice is still invariant to microstructure and shape reconfiguration as long as the Willis springs and their connections remain the same, since the elastic energy of each of the Willis springs remains unchanged, and the net torques due to reconfiguration are balanced by reaction torques from rotation-suppressed masses.

### GMMs with mapping-invariant displacement fields

**Fig. 2:**
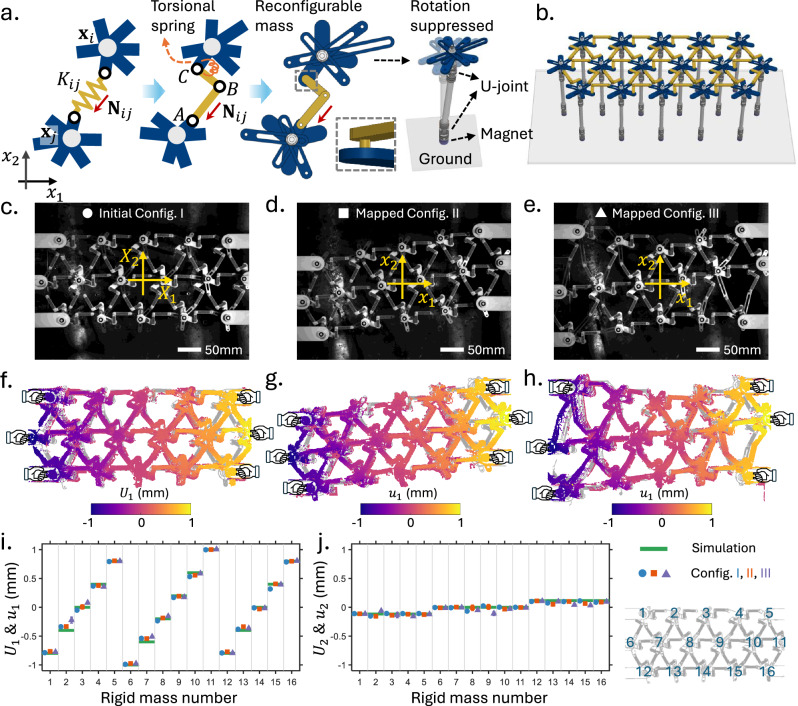
GMMs with mapping-invariant displacement fields. **a** Design of the linkage-based linear spring and the rotation-suppressed reconfigurable mass. **b** Design of the GMM composed of linkage-based linear springs and rotation-suppressed reconfigurable masses. **c**–**e** Fabricated GMM with different microstructure and shape configurations: configuration I. Rectangle (**c**); configuration II. Parallelogram (**d**); configuration III. A random shape (**e**). Experimentally measured displacement fields *u*_1_ of the three configurations under the same tensile test (deformation of the lattices has been properly scaled for better illustration): **f** configuration I; **g** configuration II; **h** configuration III. The displacement fields of the three configurations are almost the same with respect to the material coordinate, and pulling the GMMs of different shapes behaves the same as pulling a rectangular material. Experimentally measured and numerically simulated displacements *u*_1_ (**i**) and *u*_2_ (**j**) at the centers of the reconfigurable masses from repeated tensile tests. Error bars represent the standard deviation (SD) of three repeated measurements. Source data are provided as a Source data file.

We first examine the mapping-invariant displacement field by realizing and fabricating a GMM composed of linear springs and rotation-suppressed rigid masses as that shown in Fig. 1d. We then measure its displacement fields of different configurations under uniaxial tension (see Fig. 2). The linear spring is realized by a pin-connected two-bar linkage ABC connected to a fixed arm of one rigid mass through a torsional spring on joint *C* (see Fig. 2a). The torsional spring is achieved by an elastic cylinder bonded between the two-bar linkage and the rigid mass, resisting their relative rotation. As a result, when the distance between nodes *A* and *C* changes, the bar *BC* rotates, inducing forces on nodes *A* and *C*. As shown in the figure, the linkage *ABC* also attaches to a grooved movable bar of another rigid mass through a joint. Thus, the induced force is along the direction of bar *AB*, which is also the direction of the effective linear spring **N**_*ij*_. Further, by loosening the screw on the mass and adjusting the position of the grooved bar connected to the two-bar linkage, this lattice structure allows substantial microstructure and shape reconfiguration while maintaining the same spring constant and direction. Finally, to suppress mass rotation while allowing in-plane translation, we connect the reconfigurable mass to the ground through two universal joints. Note also that a permanent magnet disk is embedded between the universal joint and ground so that the GMM is able to reconfigure while simultaneously sustaining a grounded connection during deformation. Figure 2b shows the design of the GMM of a standard triangular lattice in the configuration **X**_*i*_ (see Supplementary Note 1 for design and fabrication details).

Next, the metamaterial is configured into three mapping schemes for testing: I. Rectangle; II. Parallelogram; and III. Random scheme (see Fig. 2c–e, respectively). We apply displacement boundary conditions on the left and right edges to stretch the metamaterial along the *x*_1_ direction. The displacement fields are measured using a DIC system (see Supplementary Note 2 for detailed experimental setup). Figure 2f–h shows the measured displacement fields *u*_1_ of the three configurations under the same tensile test. It can be clearly seen that the displacement fields of the three configurations are almost the same with respect to the material coordinate, and pulling the GMMs of different configurations behaves the same as pulling a rectangular material. Note that this behavior cannot be observed in free-standing materials. For example, pulling a parallelogram material usually induces bending deformation, which is shown to be absent in the properly designed and fabricated GMM. To quantify the precision of the mapping-invariant displacement field, Fig. 2i, j shows the displacements *u*_1_ and *u*_2_ at the centers of the reconfigurable masses from three repeated tensile tests. In the figures, the horizontal axis represents the sequential number of the reconfigurable mass, and the vertical axis represents the value of the displacement. It can be found that both *u*_1_ and *u*_2_ in each of the masses are nearly the same among the three configurations, and the deviations of different sets of tests are within 5%. The displacements also agree very well with the numerical results displayed by the solid lines in the figures. The mapping-invariant displacement field is also present under other types of loading, i.e., bending and shearing (see Supplementary Figs. 4 and 5 for details).

### GMMs with customized displacement fields

**Fig. 3:**
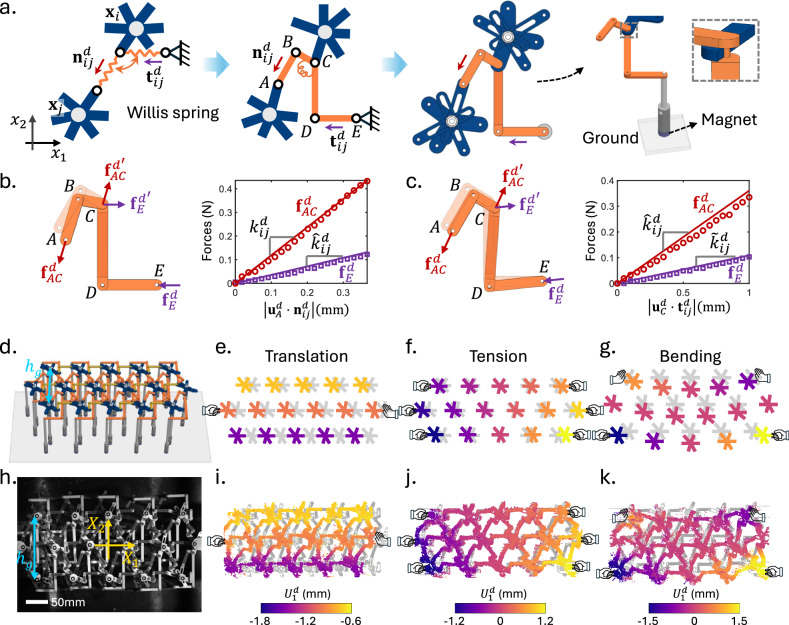
GMMs with customized displacement fields. **a** Design of the linkage-based Willis spring. **b** Experimentally measured internal and grounded forces in response to the changes in length of the internal spring. **c** Experimentally measured internal and grounded forces in response to the changes in length of the grounded spring. **d** Design of the GMM by implementing the displacement gauge $${{{{\bf{B}}}}}^{T}=[\begin{array}{cc}a & 0\\ 0 & 1\end{array}]$$. Simulated displacement fields along *X*_1_ direction when the GMM is prescribed with rigid-body translation (**e**), uniaxial tension (**f**), and pure bending (**g**). **h** Fabricated GMM based on the design in (**d**). Experimentally measured displacement fields along *X*_1_ direction when the fabricated GMM is prescribed with rigid-body translation (**i**), uniaxial tension (**j**), and pure bending (**k**). Deformation of the lattices has been properly scaled for better illustration. Source data are provided as a Source data file.

To demonstrate the capability of the GMM in achieving customized displacement fields, we design a grounded lattice composed of Willis springs and rotation-suppressed reconfigurable masses, as shown in Fig. 1g. We first realize the Willis spring described by Eqs. 1 and 2 by designing a four-bar linkage shown in Fig. 3a. In the design, the bars *BC* and *CD* are connected by a torsional spring realized by an elastic cylinder at node *C*. The other two bars are joined perpendicularly with *BC* and *CD* using pin connections. Note that the two-force member *AB* is along the internal spring direction ${{{{\bf{n}}}}}_{ij}^{d}$, and another two-force member *DE* is along the grounded spring direction ${{{{\bf{t}}}}}_{ij}^{d}$, where nodes *A* and *C* are connected between the two masses, and node *E* is connected to the ground. Clearly, when the distance between nodes *A* and *C* changes along the internal spring direction ${{{{\bf{n}}}}}_{ij}^{d}$ (where node *C* is fixed), the bar *BC* rotates, producing torques on bars *BC* and *CD*. To balance the torques, two force couples are induced, ${{{{\bf{f}}}}}_{AC}^{d}$ on nodes *A* and *C*, and ${{{{\bf{f}}}}}_{E}^{d}$ on nodes *C* and *E* (see Fig. 3b). Thus, a change in length of the internal spring generates the internal and grounded forces, ${{{{\bf{f}}}}}_{AC}^{d}$ and ${{{{\bf{f}}}}}_{E}^{d}$ along ${{{{\bf{n}}}}}_{ij}^{d}$ and ${{{{\bf{t}}}}}_{ij}^{d}$ directions, respectively. Note that $\frac{| {{{{\bf{f}}}}}_{AC}^{d}| }{| {{{{\bf{f}}}}}_{E}^{d}| }=\frac{{L}_{CD}}{{L}_{BC}}$, where *L*_*CD*_ and *L*_*BC*_ are the lengths of the bars *CD* and *BC*. Similarly, when nodes *A* and *C* move together that changes the length of the grounded spring, the bar *CD* rotates relatively to the bar *BC*, producing the two force couples ${{{{\bf{f}}}}}_{AC}^{d}$ and ${{{{\bf{f}}}}}_{E}^{d}$ in the internal and grounded springs (see Fig. 3c). Together, the internal and grounded forces read (see “Methods” and Supplementary Note 5 for details)

3 $${{{{\bf{f}}}}}_{AC}^{d}=\left[\frac{{G}^{d}({{{{\bf{u}}}}}_{A}^{d}-{{{{\bf{u}}}}}_{C}^{d})\cdot {{{{\bf{n}}}}}_{ij}^{d}}{{L}_{BC}^{2}}-\frac{{G}^{d}{{{{\bf{u}}}}}_{C}^{d}\cdot {{{{\bf{t}}}}}_{ij}^{d}}{{L}_{BC}{L}_{CD}}\right]{{{{\bf{n}}}}}_{ij}^{d},$$

4 $${{{{\bf{f}}}}}_{E}^{d}=\left[\frac{{G}^{d}({{{{\bf{u}}}}}_{A}^{d}-{{{{\bf{u}}}}}_{C}^{d})\cdot {{{{\bf{n}}}}}_{ij}^{d}}{{L}_{BC}{L}_{CD}}-\frac{{G}^{d}{{{{\bf{u}}}}}_{C}^{d}\cdot {{{{\bf{t}}}}}_{ij}^{d}}{{L}_{CD}^{2}}\right]{{{{\bf{t}}}}}_{ij}^{d},$$

where ${{{{\bf{u}}}}}_{A}^{d}$ and ${{{{\bf{u}}}}}_{C}^{d}$ are the displacements at nodes *A* and *C*, and *G*^*d*^ represents the effective torsional stiffness of the elastic cylinder. Comparing Eqs. 3 and 4 with Eqs. 1 and 2, we find that the Willis spring can be realized using the four-bar linkage by enforcing $\frac{{L}_{CD}}{{L}_{BC}}=\frac{| {{{{\bf{B}}}}}_{j}{{{{\bf{n}}}}}_{ij}| }{| ({{{{\bf{B}}}}}_{i}-{{{{\bf{B}}}}}_{j}){{{{\bf{n}}}}}_{ij}| }$ and $\frac{{G}^{d}}{{L}_{BC}^{2}}={k}_{ij}^{d}$ so that ${k}_{ij}^{d}$, ${\widehat{k}}_{ij}^{d}$, and ${\widetilde{k}}_{ij}^{d}$ can be satisfied automatically. The linkage-based spring design is experimentally validated by measuring the internal and grounded forces, ${{{{\bf{f}}}}}_{AC}^{d}$ and ${{{{\bf{f}}}}}_{E}^{d}$, in response to the changes in lengths of the internal (see Fig. 3b) and grounded springs (see Fig. 3c). The effective spring constants of the internal and grounded springs as well as the Willis coupling coefficient can be evaluated using the slopes of those lines, which agree very well with numerical results (see Supplementary Notes 3 and 6 for details).

Next, we experimentally demonstrate customized displacement fields by considering a displacement gauge $${{{{\bf{B}}}}}^{T}=[\begin{array}{cc}a & 0\\ 0 & 1\end{array}]$$, where $a=1+\frac{2{X}_{2}}{3{h}_{g}}$ with *h*_*g*_ being the height of the GMM. In this example, the displacement along *X*_1_ direction is modulated in function of *X*_2_ as ${U}_{1}^{d}=\frac{{U}_{1}}{1+\frac{2{X}_{2}}{3{h}_{g}}}$, and the displacement along *X*_2_ direction remains unchanged ${U}_{2}^{d}={U}_{2}$. By implementing the displacement gauge into Eqs. 1–4, we design and fabricate the GMM shown in Fig. 3d, h (see Supplementary Note 7 for details). Figure 3i–k show the experimentally measured displacement along the *X*_1_ direction when the GMM is prescribed with rigid-body translation, uniaxial tension, and bending, respectively. The corresponding numerical results are shown in Fig. 3e–g. As shown in Fig. 3e, i, when we push the metamaterial to the left through the two central points on the left and right boundaries, the metamaterial displays a combination of rigid-body translation and shear deformation. This can be understood by the application of the displacement gauge that introduces a bias on ${U}_{1}^{d}$ along *X*_2_ direction, which is uniform in free-standing materials and GMMs with only rotational constraints, so that the top layer of the metamaterial moves less to the left, while the bottom layer moves twice as much as the displacement on the top layer. Note that the rigid-body translation of the GMM in Fig. 3e, i costs zero elastic energy, as that in the free-standing materials, due to transformation equivalence. Thus, the deformation shown in Fig. 3e, i is a zero-energy mode. In Fig. 3f, j, we stretch the GMM along the *X*_1_ direction by enforcing displacement boundary conditions on all nodes of the left and right boundaries. It can be seen that the top layer hosts less tension than the bottom layer due to the bias of the displacement gauge. However, bending deformation, which usually appears under this type of loading, is absent in the GMM. Thus, the GMM supports asymmetric tension without bending. Finally, we apply displacements on all nodes of the left and right boundaries to bend the GMM (see Fig. 3g, k). Again, the boundary displacement applied is biased by the displacement gauge. It can be found that the vertical cross-section pointing to the *X*_1_ direction becomes nonplanar after the deformation, and the bending curvatures of the three layers of the GMM are no longer the same. The vertical cross-section and bending curvatures of longitudinal fibers can be independently controlled by the displacement gauge in the GMM. All displacement fields measured from experiments are very close to the desired fields of the design and agree well with numerical simulations. The nonstandard displacement and deformation control functions can be attributed to the embodied linkage-based Willis springs with grounded pin connections.

### GMMs with customized mapping-invariant displacement fields

**Fig. 4:**
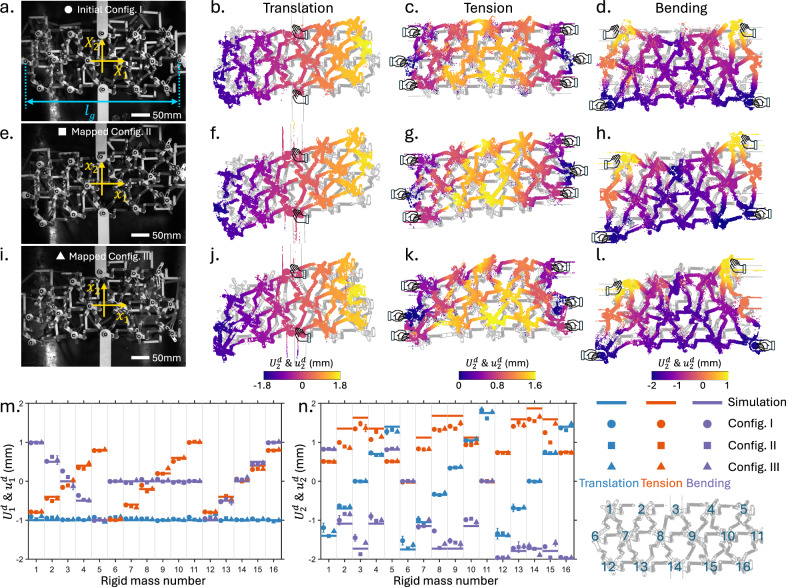
GMMs with customized mapping-invariant displacement fields. **a** Fabricated GMM (Configuration I) composed of linkage-based Willis springs and rotation-constrained reconfigurable masses, where the displacement gauge $${{{{\bf{B}}}}}^{T}=[\begin{array}{cc}1 & 0\\ -b & 1\end{array}]$$. Experimentally measured displacement along *X*_2_ direction when the GMM is prescribed with rigid-body translation (**b**), uniaxial tension (**c**), and pure bending (**d**). **e** The fabricated GMM is reconfigured to a parallelogram shape (configuration II). Experimentally measured displacement along *X*_2_ direction when the GMM is prescribed with the rigid-body translation (**f**), uniaxial tension (**g**), and pure bending (**h**). **i** The GMM is reconfigured to a random shape (configuration III). Experimentally measured displacement along *X*_2_ direction when the GMM is prescribed with the rigid-body translation (**j**), uniaxial tension (**k**), and pure bending (**l**). Deformation of the lattices has been properly scaled for better illustration. Experimentally measured displacements along *X*_1_ (**m**) and *X*_2_ (**n**) directions on the mass centers of the GMM in three configurations. Error bars represent the standard deviation (SD) of three repeated measurements. Source data are provided as a Source data file.

To further demonstrate the potential of displacement control and its mapping-invariant property, we design and fabricate another GMM by implementing a displacement gauge $${{{{\bf{B}}}}}^{T}=[\begin{array}{cc}1 & 0\\ -b & 1\end{array}]$$, where $b=-\frac{7{X}_{1}}{2{l}_{g}}$ with *l*_*g*_ being the length of the GMM (see Fig. 4a and Supplementary Note 9 for details). Using this gauge, the displacement ${U}_{2}^{d}$ is a function of the displacement *U*_1_ as ${U}_{2}^{d}=-\frac{7{X}_{1}}{2{l}_{g}}{U}_{1}+{U}_{2}$, and ${U}_{1}^{d}={U}_{1}$. We show the experimentally measured displacement along the *X*_2_ direction in Fig. 4b–d when the GMM is prescribed with rigid-body translation, uniaxial tension, and pure bending, respectively. As shown in Fig. 4b, when the GMM is moved to the left by pushing the two central masses on the top and bottom boundaries, the metamaterial is tilted and sheared with respect to the *X*_2_ direction. This is because, for the rigid-body translation, *U*_1_ is a constant, and *U*_2_ is zero; thus, ${U}_{2}^{d}$ is a linear function of *X*_1_. Note that this deformation is a zero-energy mode of the GMM. Next, when the GMM is subjected to uniaxial tension, the metamaterial is bent upward (see Fig. 4c). Under uniaxial tension, *U*_1_ is antisymmetric with respect to the *X*_2_ axis. As a result, ${U}_{2}^{d}$ is a symmetric function. Since the vertical displacements on the left and right boundaries are suppressed, the middle portion of the metamaterial is pushed up. It can also be found that ${U}_{2}^{d}$ is not uniform along the *X*_2_ direction as the bending is coupled with lateral contraction due to Poisson’s effect. Finally, the GMM is deformed under pure bending boundary constraints (Fig. 4d). It can be seen that each of the metamaterial layers is bent with different curvatures as programmed by the displacement gauge. Specifically, when the top layer is compressed, the application of the displacement gauge induces a positive curvature, which increases the original curvature from pure bending. On the other hand, the tensile deformation of the bottom layer reduces the original curvature from pure bending. The displacement fields shown in Fig. 4b–d also agree well with the numerical simulations (see Supplementary Figs. 10, 12 and 13). Together, the success of displacement control based on the two displacement gauges with control functions in diagonal and off-diagonal components indicates that an arbitrarily customized displacement field can be achieved at will using the GMM design.

Finally, the customized displacement field is also invariant to microstructure and geometric reconfigurations. To show this, we change the shape and remap the GMM to a parallelogram and to a random shape (see the fabricated samples in Fig. 4e, i). We measure the displacement fields under the same translation, tension, and bending deformation (see Fig. 4f–h, j–l, respectively). These contours reveal that despite the drastic differences in the lattice geometry, the displacement fields (in material coordinates) remain consistent with those of configuration I in Fig. 4a. Figure 4m, n quantitatively show the displacements along *X*_1_ and *X*_2_ directions at the centers of the reconfigurable masses, respectively. The displacements along both directions are nearly identical among the three configurations under rigid-body translation, uniaxial tension, and pure bending experiments, which also agree very well with numerical simulations (see Supplementary Figs. 10, 12 and 13 for details). The nonstandard displacements under other types of loads (i.e., rigid-body rotation and shear) are also invariant to configurations of the GMM (see Supplementary Figs. 11 and 14 for details).

## Discussion

In conclusion, this work unlocks a new application for the transformation method, expanding its utility from wave manipulation to the precise design of materials with mapping-invariant and customized static displacement fields. By introducing a grounded architecture, we decouple mechanical performance from geometric configuration, overcoming the shape-performance trade-off that has historically limited the reliability of reconfigurable systems. The grounded aspect is shown to be a fundamental physical requirement for transformation elasticity in solids, providing the necessary reaction body torques to balance nonconcurrent internal forces that arise during substantial reconfiguration. Another core technical breakthrough is the mechanical realization of the static Willis spring–a four-bar linkage that achieves desired Willis coupling in the static limit (*ω* → 0), a regime where such coupling traditionally vanishes. The kinematics of this spring design automatically satisfy the complex material parameter requirements of transformation elasticity, enabling nonstandard deformation modes that are forbidden in classical elasticity, such as asymmetric tension without bending and tension-induced buckling. We fabricated these metamaterials and experimentally validated their mapping invariance and nonstandard displacement control functions with appealing examples not seen before. GMMs allow the design of highly reconfigurable and efficient deformation-tailored material systems.

It should be noted that while the prototype uses magnets and rods for concept demonstrations, the theoretical framework proposed in the manuscript is scalable and offers substantial impact across multiple engineering domains. For example, in aerospace and soft robotics, GMMs enable morphing structures and sensing “skins” that maintain calibrated performance regardless of their posture or configuration. Furthermore, integrating speakers or other active components on the top surfaces of the mass nodes in GMMs allows for tunable acoustic and electromagnetic manipulation through customized mechanical deformation, enabling reconfigurable phase-modulating devices. Finally, the GMMs can be applied to support telescope mirrors to allow reconfiguration and, at the same time, to maintain position accuracy. They can also be employed for optimizing mirror focus through customized mechanical deformation in photothermal power plants.

Further, the assembled approach used for our prototype may not be suitable for small-scale applications. To assist future manufacturing, the hinges can be replaced by flexural or compliant joints (i.e., using fabric or diamond-shaped bars with small tip connections). Electrostatic or magnetic grounding can also be utilized to provide the necessary body torques and reaction forces. Thus, the entire GMM can be fabricated via monolithic fabrication processes, such as multi-material 3D printing. This fabrication method would eliminate the need for assembly. While our current prototype uses manual screws for reconfiguration, this is merely a demonstration method. In advanced applications, the grooved bars and screws can be replaced by circuit-controlled devices, such as electromagnetic linear stages or pneumatic/hydraulic actuators. This would allow the GMM to undergo active and autonomous reconfiguration in response to electrical signals, thereby removing the need for manual adjustment.

## Methods

### Detailed derivations of Eqs. 3 and 4

**Fig. 5:**
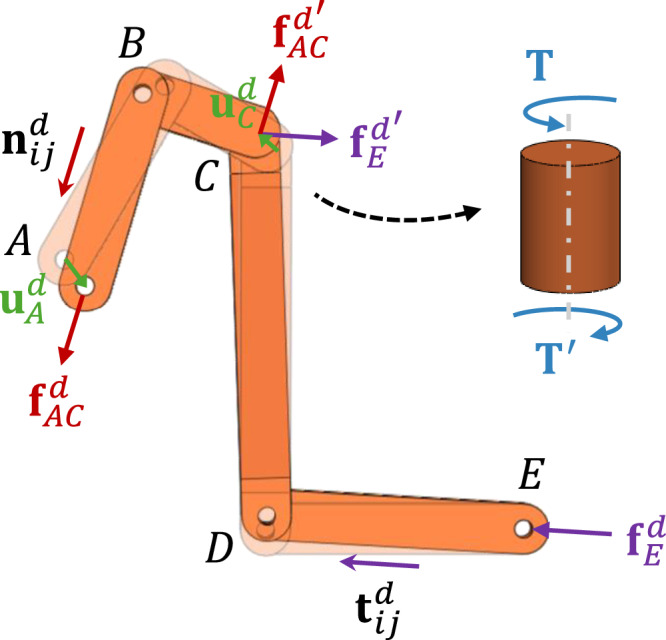
**Free-body diagram of the linkage-based Willis spring.** The schematic illustrates the internal forces and grounded forces acting on the linkage nodes, alongside the internal torques generated by the elastic cylinder during deformation. ${{\bf{f}}}_{AC}^d \,{{\rm{and}}} \,{{\bf{f}}}_{AC}^{d^\prime}$ represent the internal spring forces at nodes *A* and *C*, respectively. ${{\bf{f}}}_E^d \,{{\rm{and}}} \,{{\bf{f}}}_E^{d^\prime}$ represent the grounded spring forces at nodes *E* and *C*, respectively. ${{\bf{T}}} \,{{\rm{and}}}\, {{{\bf{T}}}^\prime}$ are the internal torques generated by the elastic cylinder. ${{\bf{n}}}_{ij}^d$ and ${{\bf{t}}}_{ij}^d$ denote the directions of the internal and grounded springs. ${{\bf{u}}}_A^d \,{{\rm{and}}} \,{{\bf{u}}}_C^d$ are the displacements at nodes *A* and *C*, respectively.

To derive Eqs. 3 and 4, we assume that the four bars in the linkage-based Willis spring are rigid. For small deformation (see Fig. 5), the kinematics of the four-bar linkage reads

5 $${{{{\bf{u}}}}}_{A}^{d}={{{{\bf{u}}}}}_{B}^{d}+{{{{\boldsymbol{\theta }}}}}_{AB}\times {{{{\bf{L}}}}}_{BA},$$

6 $${{{{\bf{u}}}}}_{B}^{d}={{{{\bf{u}}}}}_{C}^{d}+{{{{\boldsymbol{\theta }}}}}_{BC}\times {{{{\bf{L}}}}}_{CB},$$

7 $${{{{\bf{u}}}}}_{C}^{d}={{{{\bf{u}}}}}_{D}^{d}+{{{{\boldsymbol{\theta }}}}}_{CD}\times {{{{\bf{L}}}}}_{DC},$$

8 $${{{{\bf{u}}}}}_{D}^{d}={{{{\boldsymbol{\theta }}}}}_{DE}\times {{{{\bf{L}}}}}_{ED},$$

where ${{{{\bf{u}}}}}_{A}^{d}$, ${{{{\bf{u}}}}}_{B}^{d}$, ${{{{\bf{u}}}}}_{C}^{d}$, and ${{{{\bf{u}}}}}_{D}^{d}$ are the displacements at nodes *A*, *B*, *C*, and *D*, respectively, ***θ***_*AB*_, ***θ***_*BC*_, ***θ***_*CD*_ and ***θ***_*DE*_ represent the rotation vectors of bars *AB*, *BC*, *CD*, and *DE*, respectively, and **L**_*BA*_, **L**_*CB*_, **L**_*DC*_, and **L**_*ED*_ represent the length vectors of bars *AB*, *BC*, *CD*, and *DE*, respectively. Combining Eqs. 5–8 leads to

9 $$({{{{\bf{u}}}}}_{A}^{d}-{{{{\bf{u}}}}}_{C}^{d})\cdot {{{{\bf{n}}}}}_{ij}^{d}=({{{{\bf{L}}}}}_{CB}\times {{{{\bf{n}}}}}_{ij}^{d})\cdot {{{{\boldsymbol{\theta }}}}}_{BC},$$

10 $${{{{\bf{u}}}}}_{C}^{d}\cdot {{{{\bf{t}}}}}_{ij}^{d}=({{{{\bf{L}}}}}_{DC}\times {{{{\bf{t}}}}}_{ij}^{d})\cdot {{{{\boldsymbol{\theta }}}}}_{CD},$$

where ${{{{\bf{n}}}}}_{ij}^{d}=\frac{{{{{\bf{L}}}}}_{BA}}{{L}_{AB}}$, ${{{{\bf{t}}}}}_{ij}^{d}=\frac{{{{{\bf{L}}}}}_{ED}}{{L}_{DE}}$ with *L*_*AB*_ and *L*_*DE*_ being the lengths of the bars *AB* and *DE*. Since the bars *AB* and *DE* are joined using an elastic cylinder, the relative rotation of the two bars produces the internal torque, which can be written as

11 $${{{\bf{T}}}}={G}^{d}({{{{\boldsymbol{\theta }}}}}_{BC}-{{{{\boldsymbol{\theta }}}}}_{CD}),$$

where *G*^*d*^ represents the effective torsional stiffness of the elastic cylinder. To balance the torques, two force couples, ${{{{\bf{f}}}}}_{AC}^{d}$ and ${{{{\bf{f}}}}}_{E}^{d}$, are needed on nodes *A* and *C*, as well as on nodes *C* and *E*, respectively, such that ${{{\bf{T}}}}={{{{\bf{L}}}}}_{CB}\times {{{{\bf{f}}}}}_{AC}^{d}=-{{{{\bf{L}}}}}_{CD}\times {{{{\bf{f}}}}}_{E}^{d}$. Note that ${{{{\bf{f}}}}}_{AC}^{d}$ and ${{{{\bf{f}}}}}_{E}^{d}$ are the induced internal and grounded spring forces due to deformation. Combining this relationship with Eqs. 9–11, internal and grounded spring forces can be derived as

12 $${{{{\bf{f}}}}}_{AC}^{d}=\left[\frac{{G}^{d}({{{{\bf{u}}}}}_{A}^{d}-{{{{\bf{u}}}}}_{C}^{d})\cdot {{{{\bf{n}}}}}_{ij}^{d}}{{L}_{BC}^{2}}-\frac{{G}^{d}{{{{\bf{u}}}}}_{C}^{d}\cdot {{{{\bf{t}}}}}_{ij}^{d}}{{L}_{BC}{L}_{CD}}\right]{{{{\bf{n}}}}}_{ij}^{d},$$

13 $${{{{\bf{f}}}}}_{E}^{d}=\left[\frac{{G}^{d}({{{{\bf{u}}}}}_{A}^{d}-{{{{\bf{u}}}}}_{C}^{d})\cdot {{{{\bf{n}}}}}_{ij}^{d}}{{L}_{BC}{L}_{CD}}-\frac{{G}^{d}{{{{\bf{u}}}}}_{C}^{d}\cdot {{{{\bf{t}}}}}_{ij}^{d}}{{L}_{CD}^{2}}\right]{{{{\bf{t}}}}}_{ij}^{d}.$$

## Supplementary information

Supplementary Information

Transparent Peer Review File

## Source data

Source data

## Data Availability

All data needed to evaluate the conclusions in the paper are present in the paper and/or the Supplementary Information. Source data are provided with this paper.

## Code availability

The code that supports the plots of this study is provided within the Source data file.
