# Supplementary material for: Grounded reconfigurable metamaterials with customized mapping-invariant behavior
Source: Nat Commun. 2026 May 19;17:6572. doi: 10.1038/s41467-026-73240-8 (PMC13381600; doi:10.1038/s41467-026-73240-8)
Supplement: Supplementary file 1 — Supplementary Information [file 41467_2026_73240_MOESM1_ESM.pdf]

Supplementary Information for
Grounded reconfigurable metamaterials with customized mapping-invariant behavior

Yu Huang,[1] Yuxuan Tang,[1] Zhengyu Li,[1] Michael R. Haberman,[2] and Yangyang Chen[1, *]

[1]*Department of Mechanical and Aerospace Engineering, The Hong Kong University of Science and Technology, Clear Water Bay, Kowloon, Hong Kong*

[2]*Walker Department of Mechanical Engineering, University of Texas at Austin, Austin, TX 78712, USA*

Contents

Supplementary Note 1. Design and fabrication of the grounded metamaterial shown in Fig. 2c 2

Supplementary Note 2. Experimental setup for displacement field measurements 3

Supplementary Note 3. Numerical simulation details 3

Supplementary Note 4. Mapping-invariant displacement fields of bending and shearing deformation 4

Supplementary Note 5. Explanation of static Willis coupling 5

Supplementary Note 6. Experimental testing of linkage-based Willis springs 6

Supplementary Note 7. Design and fabrication of the grounded metamaterial shown in Fig. 3h 7

Supplementary Note 8. Nonstandard displacements under rigid-body rotation and shearing deformation 8

Supplementary Note 9. Design and fabrication of the grounded metamaterial shown in Fig. 4a 8

Supplementary Note 10. Nonstandard and mapping-invariant displacements of the grounded metamaterial shown in Fig. 4a 10

Supplementary Note 11. Effective Willis material properties of the grounded metamaterials 12

Supplementary References 15

*Electronic address: maeychen@ust.hk

Supplementary Note 1. DESIGN AND FABRICATION OF THE GROUNDED METAMATERIAL SHOWN IN FIG. 2C

To design the grounded metamaterial shown in Fig. 2c, we first consider a free-standing triangular virtual lattice composed of pin-connected linear springs (see Supplementary Fig. 1a). The stiffness of all springs is identical and is represented by K_0 and their free length (lattice constant) is denoted as a_0. The spring connecting nodes $\mathbf{X}_i$ and $\mathbf{X}_j$ is oriented in direction $\mathbf{N}_{ij}$. To achieve mapping-invariant displacement fields, we replace those connecting nodes by reconfigurable masses whose rigid-body rotations are suppressed, while the stiffness and orientation of the linear springs remain unchanged (see Supplementary Fig. 1b). To realize the reconfigurable masses, we design a starburst structure with six arms radiating from the attachment point. Three of the arms are made of grooved metal bars tighten to the starburst structure using the central screw that allows arbitrary shape reconfiguration (see Supplementary Fig. 1c). The linear spring is realized by a two-bar linkage bonded to the fixed arm of the starburst structure through an elastic cylinder (see Supplementary Fig. 1c). The reconfigurable mass is connected to two universal joints and a rigid bar and is attached to the ground using a permanent magnet (see Supplementary Fig. 1c). Geometric parameters of the linkage-based linear spring and the starburst structure are given in Supplementary Table 1. Supplementary Figures 1d and 1e show the fabricated grounded metamaterials, where all the white parts are 3D-printed using PLA, and the movable arms are made of aluminum alloy and fabricated using a laser cutting machine. All parts are fabricated individually and assembled together afterward to form the grounded metamaterial.

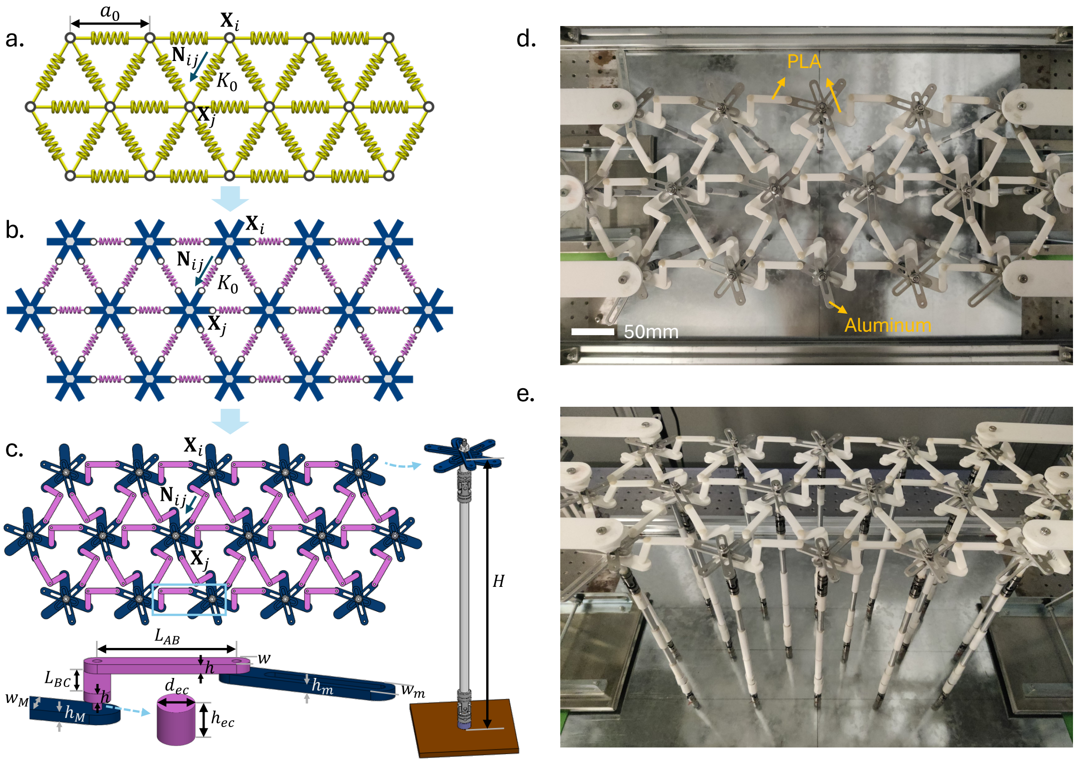

Supplementary Fig. 1: Design and fabrication of the grounded metamaterial shown in Fig. 2c. (a) A free-standing triangular lattice composed of pin-connected linear springs. (b) The grounded metamaterial composed of linear springs and rotation-suppressed reconfigurable masses. (c) Realization of the grounded metamaterial in (b). (d-e) Photographs of the fabricated grounded metamaterial.

Supplementary Table 1: Structural dimensions of the grounded metamaterial as shown in Supplementary Fig. 1.

Parameter	a_0	H	L_{AB}	L_{BC}	w	h	w_M	h_M	w_m	h_m	h_{ec}	d_{ec}
Value (mm)	90	280	36	20	7	2.5	12	2.6	9	2.5	2.5	2.5

Supplementary Note 2. EXPERIMENTAL SETUP FOR DISPLACEMENT FIELD MEASUREMENTS

The displacement fields of the grounded metamaterials are measured using the Revealer Digital Image Correlation (DIC) system (Model: RDIC-2D-DH1200; Accuracy: ≤ 0.01 pixel). Supplementary Fig. 2 shows the experimental setup of the measurement, where a camera is positioned above the metamaterial with two light sources located on the sides to offer uniform illumination. Stickers with speckle patterns are bonded to the surfaces of the grounded metamaterials to increase the accuracy of the measurement. A series of images are captured by gradually applying displacement boundary conditions through two-dimensional translation stages.

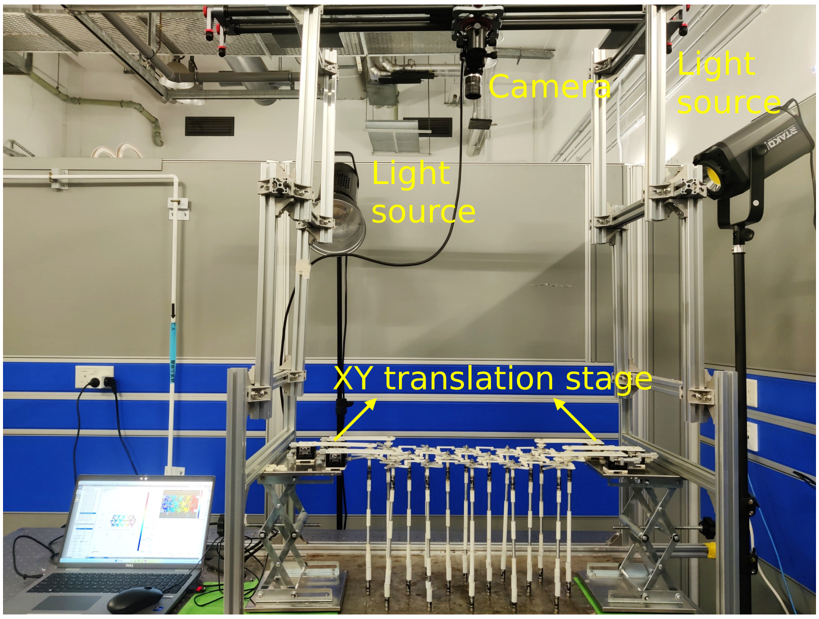

Supplementary Fig. 2: Experimental setup of the DIC system for displacement field measurements

Supplementary Note 3. NUMERICAL SIMULATION DETAILS

Numerical simulations are performed using COMSOL Multiphysics for comparison. We first simulate the displacement-force responses of the Willis spring in the form of the four-bar linkage (see Figs. 3b and 3c). 3D Structural Mechanics Module is selected, and the four-bar linkage is modeled using the geometry from fabrication. The elastic cylinder is discretized using free tetrahedral elements, while the bars are meshed using swept meshes generated from free triangular elements on their surfaces. A mesh convergence study was performed, resulting in a final mesh containing approximately 3,000 elements and 80,000 degrees of freedom. Pin connections are simulated using Rigid Connector boundary conditions. To obtain the displacement-force responses, displacement boundary conditions are applied at nodes A and C individually, and reaction forces are calculated at nodes A and E. The linear system is solved using the MUMPS direct solver. In the simulations, Young's modulus and shear modulus of PLA are selected as 1.37 GPa and 0.51 GPa, respectively. The two values are measured from three-point bending and torsion tests.

The displacement fields of the grounded metamaterial are simulated using discrete models for simplicity. The grounded metamaterial is modeled under plane stress assumptions under the Solid Mechanics interface, where only reconfigurable masses are built as solid bodies. The reconfigurable masses are meshed using triangular elements with a maximum size of $0.1a_0$. The linear and Willis springs are modeled by point forces controlled by their constitutive equations (Eqs. 3 and 4), where Point Load interfaces with functions of displacements extracted from domain point probes are implemented. Rotational degree of freedom of the reconfigurable mass is suppressed by imposing global constraints on the displacements. To simulate rigid masses, Young's modulus of the masses is set sufficiently large. The equations are solved using the MUMPS direct solver. Numerical results for Figs. 2f - 2h are shown in Supplementary Fig. 3 for comparison.

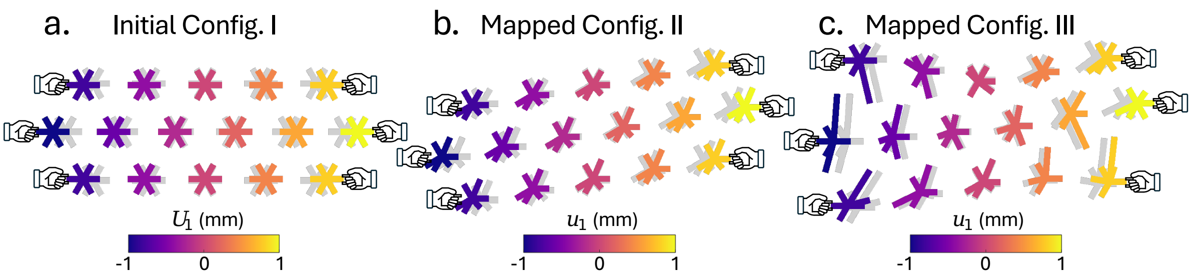

Supplementary Fig. 3: Numerical simulation results corresponding to Figs. 2f - 2h. Source data are provided as a Source Data file.

Supplementary Note 4. MAPPING-INVARIANT DISPLACEMENT FIELDS OF BENDING AND SHEARING DEFORMATION

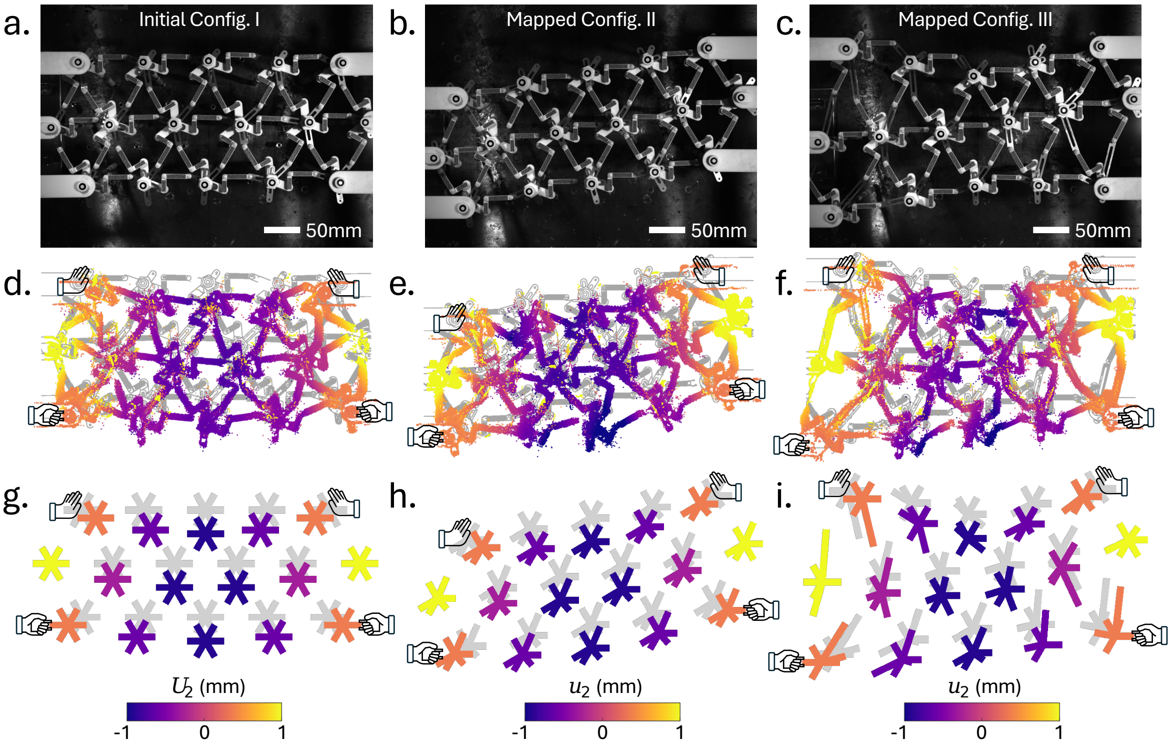

Supplementary Fig. 4: Grounded metamaterials with mapping-invariant displacement fields of bending deformation. (a-c) The metamaterial is configured in three shapes for testing: I. Rectangle (a); II. Parallelogram (b); III. A random shape (c). (d-i) Experimentally measured (d-f) and numerically simulated (g-i) displacement fields of the three configurations under the same bending test. Source data are provided as a Source Data file.

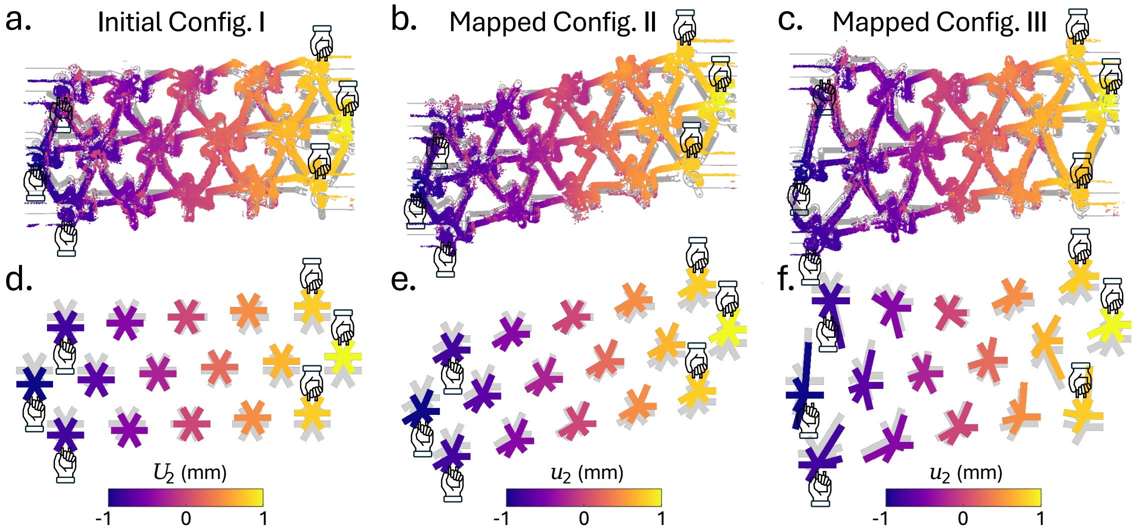

Supplementary Fig. 5: Grounded metamaterials with mapping-invariant displacement fields of shearing deformation. (a-f) Experimentally measured (a-c) and numerically simulated (d-f) displacement fields of the three configurations under the same shearing test. The three configurations are the same as those in Supplementary Fig. 4a-4c. Source data are provided as a Source Data file.

Supplementary Note 5. EXPLANATION OF STATIC WILLIS COUPLING

We provide the following points to explain the use of static Willis coupling:

- In the classical dynamic regime, Willis coupling originates from homogenization and denotes the cross-coupling between "monopole" and "dipole" quantities, i.e. between stress (dipole) and velocity (monopole), as well as between momentum (monopole) and strain (dipole) as general version of linear Cauchy elasticity. In the static limit ($\omega \to 0$), these relations undergo a mapping where velocity is replaced by displacement relative to the ground and momentum is replaced by grounded reaction forces (body forces). Thus, static Willis coupling manifests as the cross-coupling between stress and displacement, and between body force and strain, maintaining the fundamental "monopole-dipole" interaction. The reason static Willis coupling has not been widely discussed is that most traditional homogenization is performed on free-standing materials. In a free-standing material, there are no external body forces in statics, which forces the Willis coupling terms to zero. While free-standing materials can exhibit Willis coupling in the dynamic regime. Here, we can consider the inertial force as the body force. Adding asymmetry in the microstructure would couple inertial force (or body force) with spatial gradients of velocity (or strain rate), described by taking time derivative to the momentum equation in the Willis constitutive relation. Our grounded architecture provides the necessary external reference frame to host these body forces, thereby unlocking Willis coupling in the static limit.

- The substantiation of this term is further rooted in the transformation method developed in the manuscript to design GMMs. Transformation methods were previously utilized almost exclusively for wave control in optics and acoustics, where the form-invariance of governing equations is established for small-amplitude waves. However, our work unlocks a new application for the transformation method: Designing metamaterials with mapping-invariant and customized static displacement fields. Unlike wave-based transformation method, static transformation method for solid materials requires the material to be polar and exhibit the cross-coupling between stress and displacement, and between body force and strain under the coordinate transformation with nonuniform displacement gauges.

$$f_{ij}^d = [k_{ij}^d(u_j^d - u_i^d)\cdot n_{ij}^d - \hat{k}_{ij}^d u_i^d \cdot t_{ij}^d]n_{ij}^d, \tag{S1}$$

$$\bar{f}_i^d = [\hat{k}_{ij}^d(u_j^d - u_i^d)\cdot n_{ij}^d - \tilde{k}_{ij}^d u_i^d \cdot t_{ij}^d]t_{ij}^d. \tag{S2}$$

where $\hat{k}_{ij}^d$ describe the cross-coupling and is the static Willis coupling coefficient as we introduce and define above. Thus, static Willis coupling is the requirement to achieve the mapping-invariant and customized displacement fields.

- Finally, we want to highlight that not every linkage connected to the external world can realize the specific Willis coupling required by transformation elasticity. The linkage must be carefully designed to satisfy the requirement that $(\hat{k}_{ij}^d)^2 = k_{ij}^d \tilde{k}_{ij}^d$ (according to the definitions of these three coefficients), meaning the stiffness matrix of the linkage-based Willis spring must be degenerate with a rank equal to 1. To meet this stringent requirement, we designed the four-bar linkage that comprises a torsional spring. Based on the equilibrium conditions between the torques on this torsional spring, the constitutive equations of the four-bar linkage read

$$\mathbf{f}_{AC}^d = \left[\frac{G^d \left(\mathbf{u}_A^d - \mathbf{u}_C^d \right) \cdot \mathbf{n}_{ij}^d}{L_{BC}^2} - \frac{G^d \mathbf{u}_C^d \cdot \mathbf{t}_{ij}^d}{L_{BC} L_{CD}} \right] \mathbf{n}_{ij}^d, \tag{S3}$$

$$\mathbf{f}_{E}^d = \left[\frac{G^d \left(\mathbf{u}_A^d - \mathbf{u}_C^d \right) \cdot \mathbf{n}_{ij}^d}{L_{BC} L_{CD}} - \frac{G^d \mathbf{u}_C^d \cdot \mathbf{t}_{ij}^d}{L_{CD}^2} \right] \mathbf{t}_{ij}^d, \tag{S4}$$

  where $\mathbf{u}_A^d$ and $\mathbf{u}_C^d$ are the displacements at nodes A and C, and G^d represents the effective torsional stiffness of the elastic cylinder. Comparing these to the theoretical Willis relations, we find that the Willis parameters are satisfied automatically by enforcing: $\frac{L_{CD}}{L_{BC}} = \frac{|\mathbf{B}_j \mathbf{n}_{ij}|}{|(\mathbf{B}_i - \mathbf{B}_j)\mathbf{n}_{ij}|}$ and $\frac{G^d}{L_{BC}^2} = K_{ij}^d$. This automatic satisfaction via geometric ratios is a key technical contribution, as it ensures the physical hardware exactly mimics the complex cross-coupling required by the transformation method. This degeneracy is the special physical signature that substantiates the mechanism as a true carefully designed static Willis spring.

Supplementary Note 6. EXPERIMENTAL TESTING OF LINKAGE-BASED WILLIS SPRINGS

Figure 6 illustrates the experimental setup for testing the linkage-based Willis spring. Components of the linkage-based Willis spring are 3D-printed using PLA. The bars BC, CD, and the elastic cylinder at node C are printed together as a single integrated unit, with all parts subsequently assembled into the four-bar linkage to form the linkage-based Willis spring. To test the response of the linkage-based Willis spring, displacement boundary conditions are applied at nodes A and C using two-dimensional translation stages (Make: CPC; Model: LY40-LM; Accuracy of the step sizes: 10 μm). The internal and grounded forces, $\mathbf{f}_{AC}^d$ and $\mathbf{f}_E^d$, are measured at nodes A and E using three-axis force sensors (Make: Ligentcn; Model: LF-303M-40; Sensitivity: $1.0 \pm 10\%$ mV/V). To retrieve the Willis spring constants, slopes of the displacement-force response curves are measured. Good agreement between experimental and numerical results is observed.

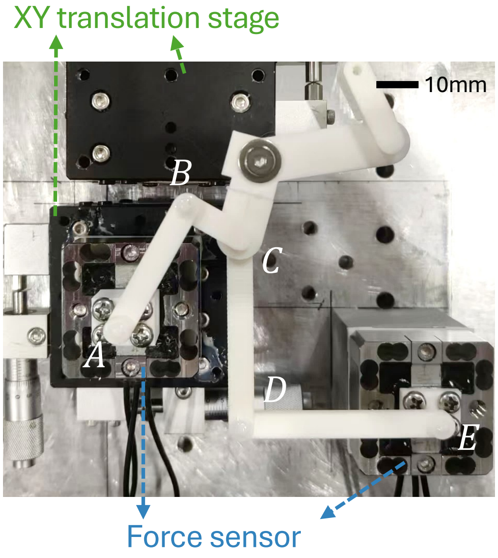

Supplementary Fig. 6: Experimental setup for testing the linkage-based Willis spring.

Supplementary Note 7. DESIGN AND FABRICATION OF THE GROUNDED METAMATERIAL SHOWN IN FIG. 3H

To design the grounded metamaterial, we select a free-standing triangular lattice with the lattice constant $a_0 = 90$ mm and $K_0 = 1.25$ N/mm (see Supplementary Fig. 7a) before the transformation. Implementing the displacement gauge $\mathbf{B}^T = \begin{bmatrix} 1+\frac{2X_2}{3h_g} & 0 \\ 0 & 1 \end{bmatrix}$ into Eqs. (1) and (2), Willis spring constants k_{ij}^d, $\hat{k}_{ij}^d$, $\tilde{k}_{ij}^d$ and the internal and grounded spring directions $\mathbf{n}_{ij}^d$ and $\mathbf{t}_{ij}^d$ are then determined, which are schematically shown in Supplementary Fig. 7b. To realize those ideal Willis springs, we next design a series of four-bar linkage structures (see Supplementary Fig. 7c). To facilitate installation, we first select the length of the bar BC as 16 mm ($L_{BC} = 16\,\mathrm{mm}$) according to the lattice constant. Based on Eqs. (3) and (4), the length of the bar CD can then be determined as $L_{CD} = \frac{L_{BC} k_{ij}^d}{\hat{k}_{ij}^d}$. The length of the bar DE L_{DE} is set to be greater than 40 mm to allow free global translation along the X_2 direction. The length of the fourth bar AB L_{AB} is determined by the its orientation $\mathbf{n}_{ij}^d$ and the length of the bar BC L_{BC}, and requires appropriate adjustment to support proper connections between fixed and movable arms. The width and thickness of all bars are 7 and 2.5 mm such that those bars can be considered rigid during deformation to satisfy theoretical assumptions. Having completed the design of the four bars, we move to the design of the elastic cylinder. The elastic cylinder is required to display an effective torsional stiffness $G^d = k_{ij}^d L_{BC}^2$. To satisfy this requirement, we first estimate the torsional stiffness of the elastic cylinder by $G^d = \frac{G_{ec} J_{ec}}{h_{ec}}$, where G_{ec}, J_{ec}, and h_{ec} denote the shear modulus, polar moment of inertia of the cross-sectional area, and height of the elastic cylinder, respectively. By selecting $h_{ec} = 3\,\mathrm{mm}$, the diameter of the elastic cylinder d_{ec} can be quickly determined. However, due to local deformation on the interfaces between the bars and the cylinder, d_{ec} is slightly adjusted according to numerical simulations and experimental measurements to precisely satisfy the required Willis spring constants. Detailed geometric parameters of the grounded metamaterial design are provided in Supplementary Table 2. Fabrication of the grounded metamaterial is similar to that demonstrated in Supplementary Note 1. Supplementary Figures 7d and 7e show the fabricated sample for experimental measurements.

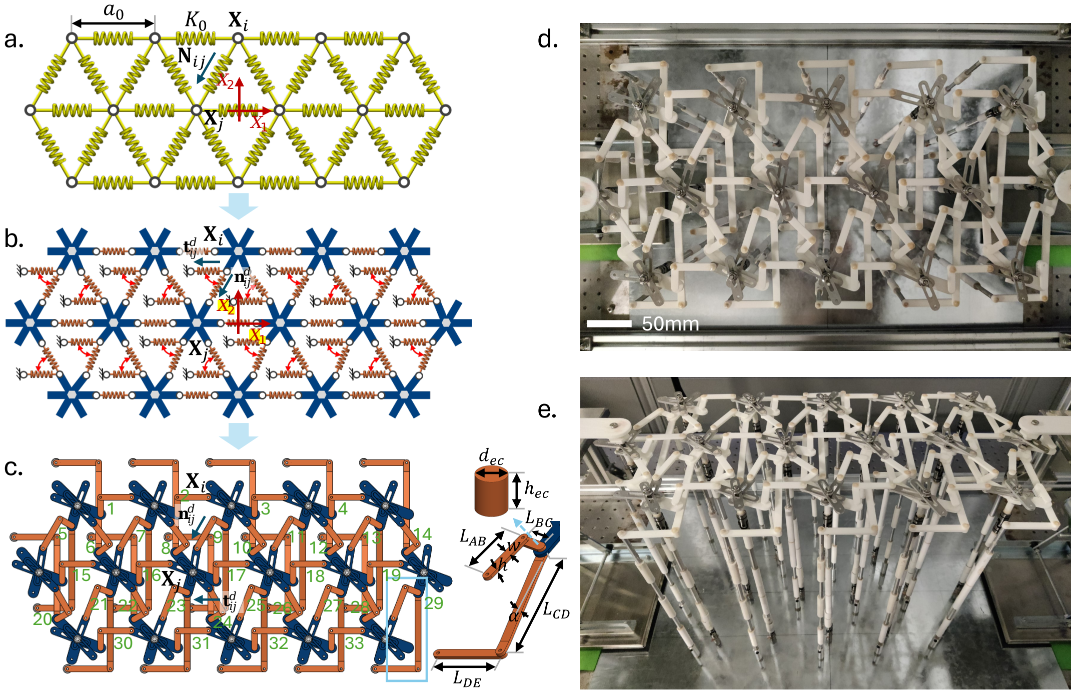

Supplementary Fig. 7: Design and fabrication of the grounded metamaterial shown in Fig. 3h. (a) A free-standing triangular lattice composed of pin-connected linear springs. (b) The grounded metamaterial composed of Willis springs and rotation-suppressed reconfigurable masses with the displacement gauge $\mathbf{B}^T = \begin{bmatrix} a & 0 \\ 0 & 1 \end{bmatrix}$. (c) Realization of the grounded metamaterial in (b). (d-e) Photographs of the fabricated grounded metamaterial.

Supplementary Table 2: Key structural dimensions of all springs in the grounded metamaterial as shown in Supplementary Fig. 7.

Spring	k_{ij}^d (N/mm)	L_{BC} (mm)	L_{CD} (mm)	α	h_{ec} (mm)	d_{ec} (mm)
1 – 4	2.22					3
15 – 19	1.25	20	—	—	2.5	2.5
30 – 33	0.44					1.96
5, 7, 9, 11, 13, 20, 22, 24, 26, 28	1.25		96.0			2.27
6, 8, 10, 12, 14	1.49	16	104.9	5°	3	2.44
21, 23, 25, 27, 29	1.08		89.1			2.22

Supplementary Note 8. NONSTANDARD DISPLACEMENTS UNDER RIGID-BODY ROTATION AND SHEARING DEFORMATION

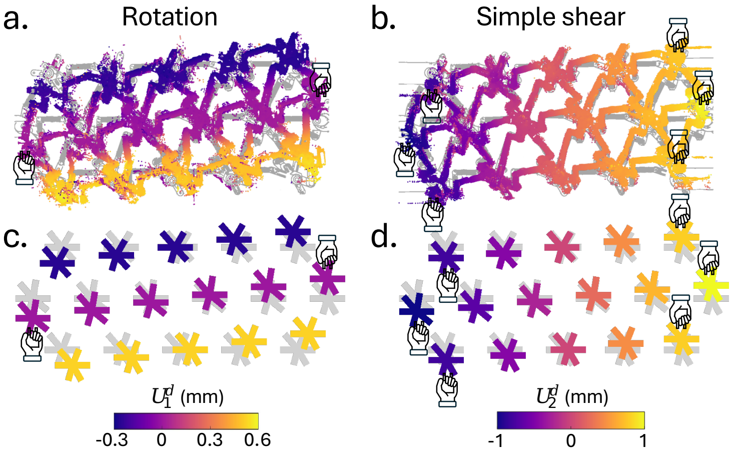

Supplementary Fig. 8: (a-d) Experimentally measured (a,b) and numerically simulated (c,d) displacement fields under rigid-body rotation and shearing deformation with displacement gauge $\mathbf{B}^T = \begin{bmatrix} a & 0 \\ 0 & 1 \end{bmatrix}$. Source data are provided as a Source Data file.

Supplementary Note 9. DESIGN AND FABRICATION OF THE GROUNDED METAMATERIAL SHOWN IN FIG. 4A

Following the design and fabrication procedures demonstrated in Supplementary Note 7, the grounded metamaterial shown in Fig. 4a is designed and fabricated (see Supplementary Fig. 9). Geometric parameters of the design are given in Supplementary Table 3.

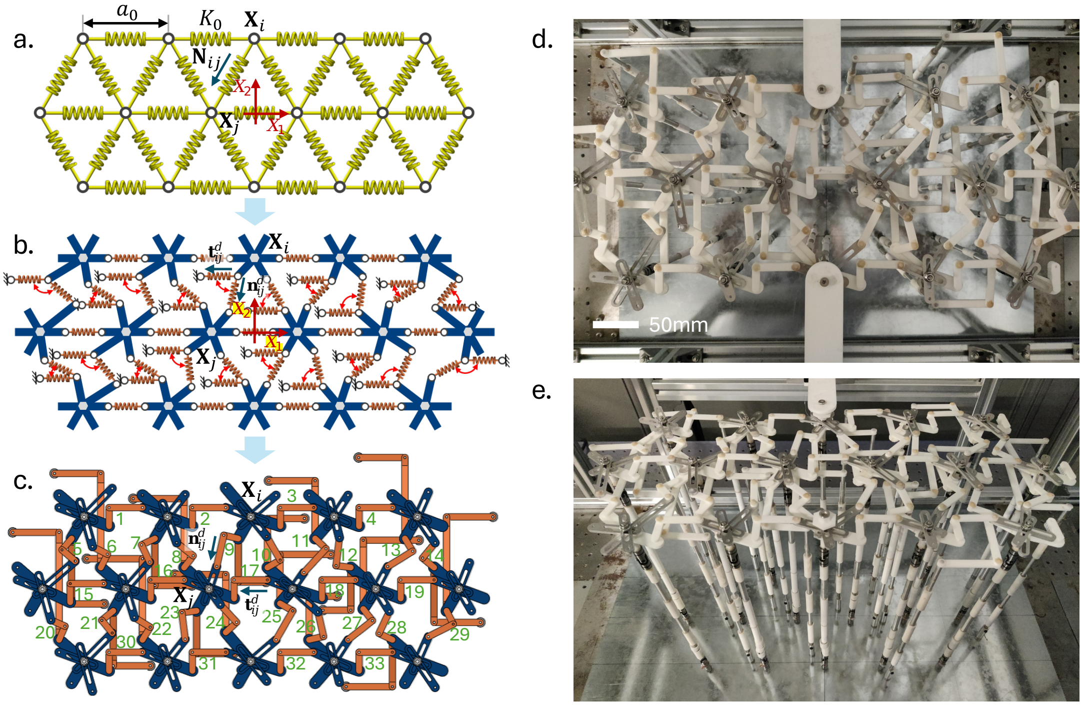

Supplementary Fig. 9: Design and fabrication of the grounded metamaterial shown in Fig. 4a. (a) A free-standing triangular lattice composed of pin-connected linear springs. (b) The grounded metamaterial composed of Willis springs and rotation-suppressed reconfigurable masses with the displacement gauge $\mathbf{B}^T = \begin{bmatrix} 1 & 0 \\ -b & 1 \end{bmatrix}$. (c) Realization of the grounded metamaterial in (b). (d-e) Photographs of the fabricated grounded metamaterial.

Supplementary Table 3: Key structural dimensions of all springs in the grounded metamaterial as shown in Supplementary Fig. 9.

Spring	k_{ij}^d (N/mm)	L_{BC} (mm)	L_{CD} (mm)	α	h_{ec} (mm)	d_{ec} (mm)
1 – 4, 15 – 19, 30 – 33	1.25	20	—	—	2.5	2.5
5	2.23		70.5			2.87
6, 29	4.61		101.3			4.5
7, 28	1.15		50.6			2.24
8, 27	2.47		74.2			3
9, 26	0.98		46.9			2.18
10, 25	1.25	16	52.8	5°	3	2.27
11, 24	1.75		62.4			2.61
12, 23	0.95		46.1			2.18
13, 22	3.42		87.3			3.6
14, 21	1.57		59.2			2.5
20	6.02		115.8			5.7

Supplementary Note 10. NONSTANDARD AND MAPPING-INVARIANT DISPLACEMENTS OF THE GROUNDED METAMATERIAL SHOWN IN FIG. 4A

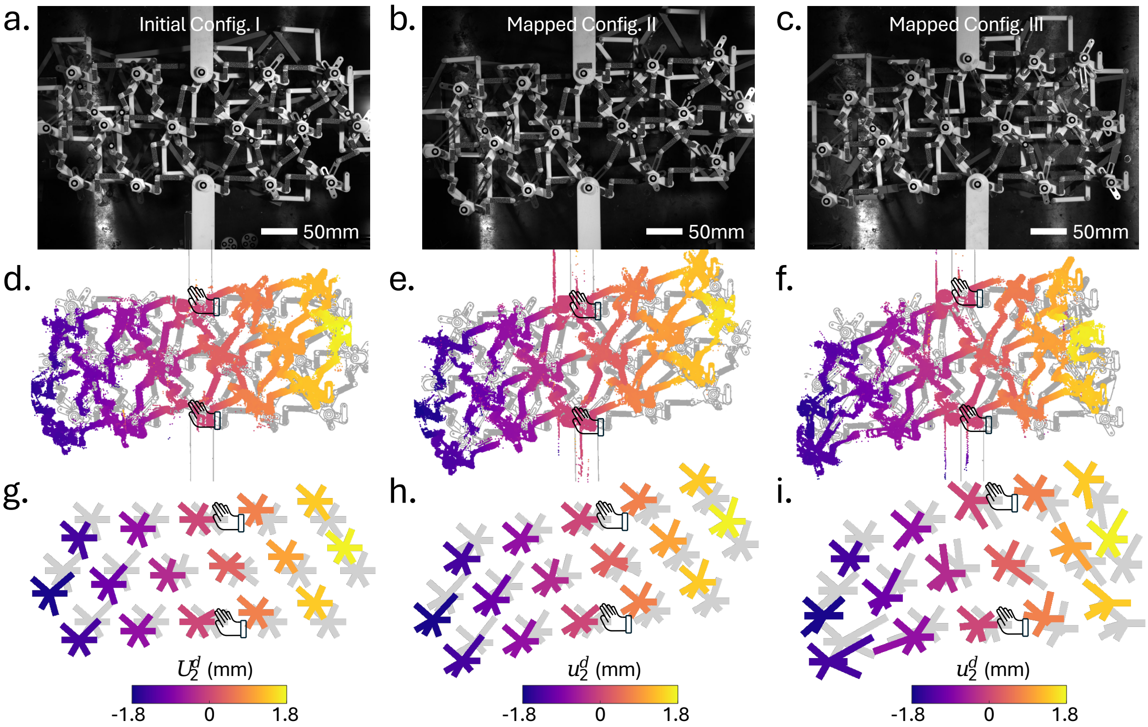

Supplementary Fig. 10: Grounded metamaterial with nonstandard and mapping-invariant displacements under rigid-body translation. (a-c) The metamaterial is configured in three shapes for testing: I. Rectangle (a); II. Parallelogram (b); III. A random shape (c). (d-i) Experimentally measured (d-f) and numerically simulated (g-i) displacement fields of the three configurations under the same translational test. Source data are provided as a Source Data file.

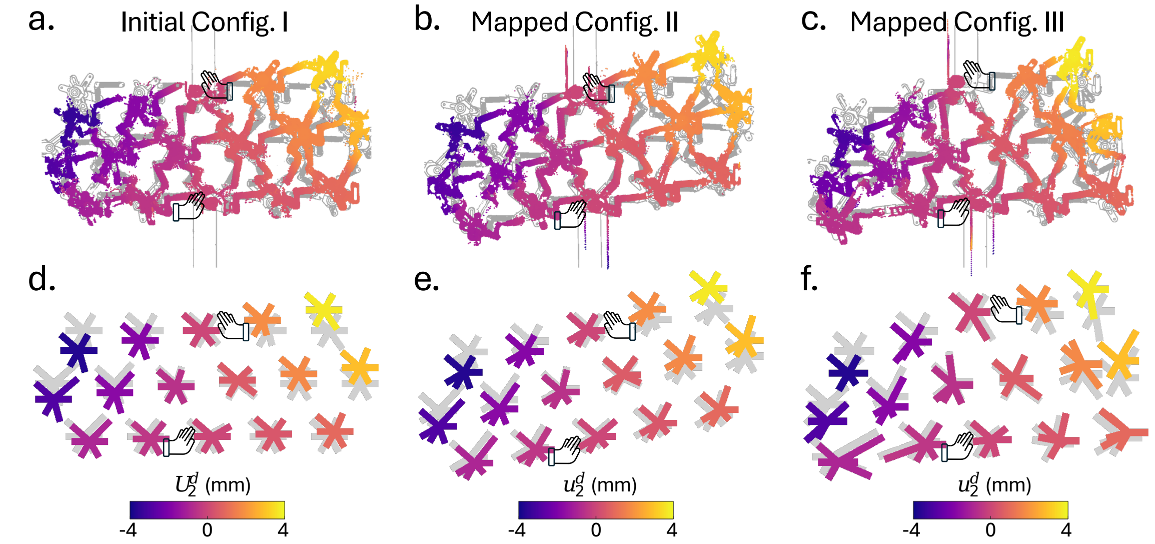

Supplementary Fig. 11: Grounded metamaterial with nonstandard and mapping-invariant displacements under rigid-body rotation. (a-f) Experimentally measured (a-c) and numerically simulated (d-f) displacement fields of the three configurations under the same rotational test. The three configurations are the same as those in Supplementary Figs. 10a-10c. Source data are provided as a Source Data file.

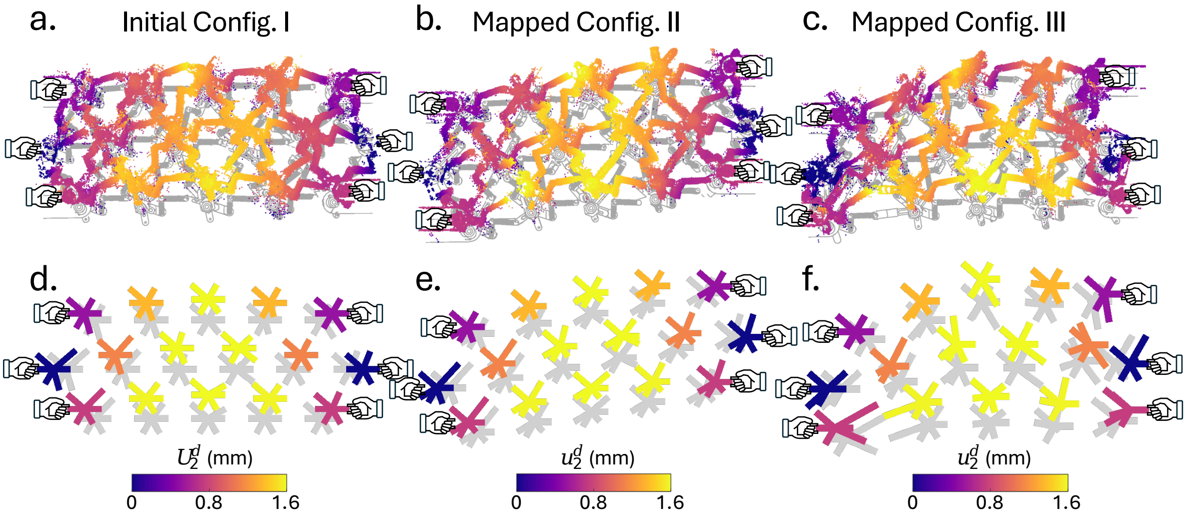

Supplementary Fig. 12: Grounded metamaterial with nonstandard and mapping-invariant displacements under tensile deformation. (a-f) Experimentally measured (a-c) and numerically simulated (d-f) displacement fields of the three configurations under the same tensile test. The three configurations are the same as those in Supplementary Figs. 10a-10c. Source data are provided as a Source Data file.

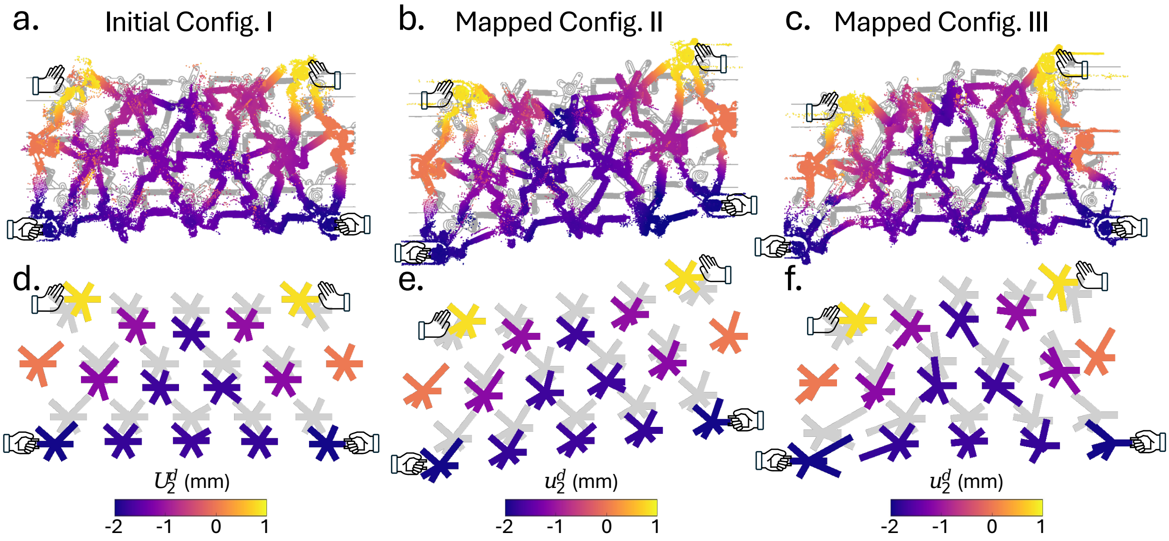

Supplementary Fig. 13: Grounded metamaterial with nonstandard and mapping-invariant displacements under bending deformation. (a-f) Experimentally measured (a-c) and numerically simulated (d-f) displacement fields of the three configurations under the same bending test. The three configurations are the same as those in Supplementary Figs. 10a-10c. Source data are provided as a Source Data file.

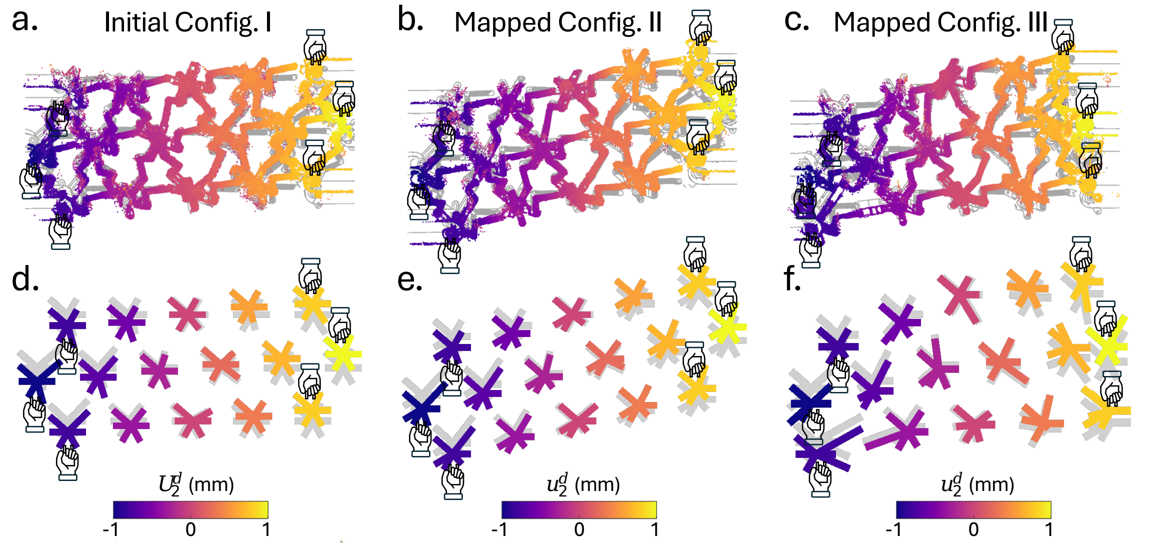

Supplementary Fig. 14: Grounded metamaterial with nonstandard and mapping-invariant displacements under shearing deformation. (a-f) Experimentally measured (a-c) and numerically simulated (d-f) displacement fields of the three configurations under the same shearing test. The three configurations are the same as those in Supplementary Figs. 10a-10c. Source data are provided as a Source Data file.

Supplementary Note 11. EFFECTIVE WILLIS MATERIAL PROPERTIES OF THE GROUNDED METAMATERIALS

We first revisit the continuum transformation elasticity and then perform homogenization for the grounded metamaterials derived from discrete transformation with different geometric mapping. Finally, we compare the effective elastic properties with those from continuum transformation.

To formulate the continuum transformation, we first consider a virtual elastic solid occupied in $\mathbf{X}$ and assume that the virtual solid obeys Hooke's law. The virtual solid deforms with the displacement field $\mathbf{U}(\mathbf{X})$ in response to a

given set of external loads or constraints. Our goal is to calculate the material properties of the transformed solid to realize the desired displacement field $\mathbf{u}(\mathbf{x})$ in a transformed coordinate $\mathbf{x}$. To facilitate the coordinate transformation, it is useful to write the elastic energy density of the virtual solid as

$$\mathcal{E} = \frac{1}{2} C_{ijkl} \frac{\partial U_j}{\partial X_i} \frac{\partial U_l}{\partial X_k}, \tag{S5}$$

where C_{ijkl} denotes the elasticity tensor of the virtual solid, and the stress tensor reads $\Sigma_{ij} = C_{ijkl} \frac{\partial U_l}{\partial X_k}$. Note that we only consider linear deformations and Cauchy elasticity. To achieve the desired displacement field $\mathbf{u}(\mathbf{x})$ in the physical solid, we introduce a displacement gauge $\mathbf{B}(\mathbf{X})$ defined as [1]

$$\mathbf{u}(\mathbf{x}) = \left(\mathbf{B}(\mathbf{X})^T\right)^{-1} \mathbf{U}(\mathbf{X}), \tag{S6}$$

where $\mathbf{B}(\mathbf{X})$ is an invertible matrix. The spatial derivative of the virtual displacement field then read

$$\frac{\partial U_j}{\partial x_i} = B_{pj} \frac{\partial u_p}{\partial x_i} + \frac{\partial X_q}{\partial x_i} \frac{\partial B_{pj}}{\partial X_q} u_p. \tag{S7}$$

Implementing coordinate transformation by substituting Eq. (S7) into Eq. (S5), we obtain the elastic energy density in $\mathbf{x}$ as

$$e = \frac{1}{2} \left(\frac{\partial u_j}{\partial x_i} \sigma_{ij} + u_k f_k \right), \tag{S8}$$

$$\sigma_{ij} = c_{ijkl} \frac{\partial u_l}{\partial x_k} + s_{ijk} u_k, \tag{S9}$$

$$f_k = s_{ijk} \frac{\partial u_j}{\partial x_i} + d_{kl} u_l, \tag{S10}$$

where $c_{ijkl} = \frac{1}{J} C_{pqst} \frac{\partial x_i}{\partial X_p} B_{jq} \frac{\partial x_k}{\partial X_s} B_{lt}$, $s_{ijk} = \frac{1}{J} C_{pqst} \frac{\partial x_i}{\partial X_p} B_{jq} \frac{\partial B_{kt}}{\partial X_s}$, $d_{kl} = \frac{1}{J} C_{pqst} \frac{\partial B_{kq}}{\partial X_p} \frac{\partial B_{lt}}{\partial X_s}$ with $J = \det\left(\frac{\partial x_i}{\partial X_j}\right)$, and e, σ_{ij}, and f_k denote the elastic energy density, stress, and body force of the transformed solid, respectively. Clearly, the elastic energy density of the transformed solid e no longer keeps its original form after the transformation. Instead, it has two parts: one comes from the strain energy density; the other arises due to the work done by the body force, a new term caused by the transformation. Further, the stress-strain relationship of the transformed solid is no longer Hooke's law, as noted by inspection of Eq. (S9) and the definition of s_{ijk}, which depends on the transformation. Equation (S9) shows that stresses in the transformed solid depend on both the displacement gradients and the displacement itself through a elastostatic Willis coupling tensor s_{ijk}. In a similar manner, body forces are determined by not only the displacement but the displacement gradients through the same Willis coupling tensor s_{ijk}. Secondly, the elasticity tensor of the transformed solid loses minor symmetry, e.g. $c_{ijkl} \neq c_{ijlk} \neq c_{jilk}$, leading to asymmetric stresses, a response characterizing elastic polarity. Once the two properties are satisfied in a solid material, the desired displacement behavior will be guaranteed. The observations here are identical with that derived from the discrete transformation shown in the main text.

Next, we homogenize the GMM design in the main text and calculate its effective elasticity tensors. The potential energy per unit area is calculated under a macroscopic displacement gradient $\mathbf{e}$ and a uniform displacement $\mathbf{u}$ over a unit cell of the GMM. For the triangular lattice demonstrated in the examples, we decompose the potential energy density $\bar{\varepsilon}$ into three parts caused by the three springs $\{i,j\}$, $\{i,k\}$, and $\{j,k\}$ in the unit cell. The potential energy density then reads

$$\bar{\varepsilon} = \bar{\varepsilon}_{ij} + \bar{\varepsilon}_{ik} + \bar{\varepsilon}_{jk}, \tag{S11}$$

where

$$\bar{\varepsilon}_{ij} = \frac{k_{ij}^d}{2A} \left(\mathbf{e}\mathbf{r}_{ij}\mathbf{n}_{ij}^d\right)^2 + \frac{\hat{k}_{ij}^d}{A} \left(\mathbf{e}\mathbf{r}_{ij}\mathbf{n}_{ij}^d\right)\left(\mathbf{u}\mathbf{t}_{ij}^d\right) + \frac{\tilde{k}_{ij}^d}{2A} \left(\mathbf{u}\mathbf{t}_{ij}^d\right)^2, \tag{S12}$$

$$\mathbf{r}_{ij} = \mathbf{x}_i - \mathbf{x}_j, \tag{S13}$$

and A is the area of the unit cell. The effective elasticity tensors of the GMM can be derived by

$$\bar{c}_{ijkl} = \frac{\partial^2 \bar{\varepsilon}}{\partial e_{ij} \partial e_{kl}}, \tag{S14}$$

$$\bar{s}_{ijk} = \frac{\partial^2 \bar{\varepsilon}}{\partial e_{ij} \partial u_k}, \tag{S15}$$

$$\bar{d}_{ij} = \frac{\partial^2 \bar{\varepsilon}}{\partial u_i \partial u_j}. \tag{S16}$$

Calculating Eqs. (S14-S16) at the continuum limit, the effective elasticity tensors are equal to those derived from continuum transformation, $\bar{c}_{ijkl} = c_{ijkl}$, $\bar{s}_{ijk} = s_{ijk}$, and $\bar{d}_{ij} = d_{ij}$.

Next, we validate these relationships through two illustrative examples by calculating effective elastic properties using continuum transformation elasticity and performing homogenization for the grounded metamaterials. We assume a free-standing triangular lattice in the virtual space (as shown in the main text) with uniform spring constant K_0. Its effective elasticity tensor reads

$$\mathbf{C} = \begin{bmatrix} \lambda + 2\mu & \lambda & 0 & 0 \\ \lambda & \lambda + 2\mu & 0 & 0 \\ 0 & 0 & \mu & \mu \\ 0 & 0 & \mu & \mu \end{bmatrix}, \tag{S17}$$

where $\lambda = \mu = \frac{\sqrt{3}K_0}{4}$.

Example I:

We select the displacement gauge $\mathbf{B} = \begin{bmatrix} 1 & -b(X_1) \\ 0 & 1 \end{bmatrix}$ and $\mathbf{x} = \mathbf{X}$ corresponding to the example demonstrated in Fig. 4a. Implementing this displacement gauge and coordinate mapping into Eqs. (S8-S10), transformed elasticity tensors derived from continuum transformation can be written as

$$\mathbf{c} = \mu \begin{bmatrix} 3 + b^2 & 1 & -b & -2b \\ 1 & 3 & 0 & -3b \\ -b & 0 & 1 & 1 \\ -2b & -3b & 1 & 1 + 3b^2 \end{bmatrix}, \tag{S18}$$

$$\mathbf{s} = \mu \begin{bmatrix} b\frac{\partial b}{\partial X_1} & 0 \\ 0 & 0 \\ -\frac{\partial b}{\partial X_1} & 0 \\ -\frac{\partial b}{\partial X_1} & 0 \end{bmatrix}, \tag{S19}$$

$$\mathbf{d} = \mu \begin{bmatrix} (\frac{\partial b}{\partial X_1})^2 & 0 \\ 0 & 0 \end{bmatrix}. \tag{S20}$$

Next, we perform homogenization for the grounded metamaterial shown in Fig. 4a based on Eqs. (S11-S16), the effective elasticity tensors are attained as

$$\bar{\mathbf{c}} = \frac{\sqrt{3}K_0}{4} \begin{bmatrix} 3 + b_j^2 & 1 & -b_j & -2b_j \\ 1 & 3 & 0 & -3b_j \\ -b_j & 0 & 1 & 1 \\ -2b_j & -3b_j & 1 & 1 + 3b_j^2 \end{bmatrix}, \tag{S21}$$

$$\bar{\mathbf{s}} = \frac{\sqrt{3}K_0}{4} \begin{bmatrix} \frac{2(b_j - b_k)}{a_0} b_j & 0 \\ 0 & 0 \\ -\frac{2(b_j - b_k)}{a_0} & 0 \\ -\frac{2(b_j - b_k)}{a_0} & 0 \end{bmatrix}, \tag{S22}$$

$$\bar{\mathbf{d}} = \frac{\sqrt{3}K_0}{4}\begin{bmatrix} \frac{4(b_j-b_k)^2}{a_0^2} & 0 \\ 0 & 0 \end{bmatrix}, \tag{S23}$$

where b_j denotes $b(\mathbf{X}_j)$. Comparing Eqs. (S21-S23) with Eqs. (S18-S20), it can be clearly seen that $\bar{\mathbf{c}} = \mathbf{c}$, $\bar{\mathbf{s}} = \mathbf{s}$, and $\bar{\mathbf{d}} = \mathbf{d}$ at the continuum limit.

Example II:

In the second example, we consider the parallelogram mapping corresponding to the grounded metamaterial in Fig. 4e, where $\nabla_X \mathbf{x} = \begin{bmatrix} 1 & 0 \\ \gamma & 1 \end{bmatrix}$, γ is a constant. Again, the transformed elasticity tensors derived from continuum transformation can be written as

$$\mathbf{c} = \mu \begin{bmatrix} 3+b^2 & 1-\gamma b & -b & 3\gamma-2b+\gamma b^2 \\ 1-\gamma b & 3+\gamma^2 & \gamma & 2\gamma-3b-\gamma^2 b \\ -b & \gamma & 1 & 1-\gamma b \\ 3\gamma-2b+\gamma b^2 & 2\gamma-3b-\gamma^2 b & 1-\gamma b & 1+3\gamma^2-4\gamma b+3b^2+\gamma^2 b^2 \end{bmatrix}, \tag{S24}$$

$$\mathbf{s} = \mu \begin{bmatrix} b\frac{\partial b}{\partial X_1} & 0 \\ -\gamma\frac{\partial b}{\partial X_1} & 0 \\ -\frac{\partial b}{\partial X_1} & 0 \\ (\gamma b-1)\frac{\partial b}{\partial X_1} & 0 \end{bmatrix}, \tag{S25}$$

$$\mathbf{d} = \mu \begin{bmatrix} (\frac{\partial b}{\partial X_1})^2 & 0 \\ 0 & 0 \end{bmatrix}. \tag{S26}$$

The homogenized elasticity tensors read

$$\bar{\mathbf{c}} = \frac{\sqrt{3}K_0}{4}\begin{bmatrix} 3+b_j^2 & 1-\gamma b_j & -b_j & 3\gamma-2b_j+\gamma b_j^2 \\ 1-\gamma b_j & 3+\gamma^2 & \gamma & 2\gamma-3b_j-\gamma^2 b_j \\ -b_j & \gamma & 1 & 1-\gamma b_j \\ 3\gamma-2b_j+\gamma b_j^2 & 2\gamma-3b_j-\gamma^2 b_j & 1-\gamma b_j & 1+3\gamma^2-4\gamma b_j+3b_j^2+\gamma^2 b_j^2 \end{bmatrix}, \tag{S27}$$

$$\bar{\mathbf{s}} = \frac{\sqrt{3}K_0}{4}\begin{bmatrix} \frac{2(b_j-b_k)}{a_0}b_j & 0 \\ -\frac{2(b_j-b_k)}{a_0}\gamma & 0 \\ -\frac{2(b_j-b_k)}{a_0} & 0 \\ \frac{2(b_j-b_k)}{a_0}(\gamma b_j-1) & 0 \end{bmatrix}, \tag{S28}$$

$$\bar{\mathbf{d}} = \frac{\sqrt{3}K_0}{4}\begin{bmatrix} \frac{4(b_j-b_k)^2}{a_0^2} & 0 \\ 0 & 0 \end{bmatrix}. \tag{S29}$$

Comparing Eqs. (S27-S29) with Eqs. (S24-S26), the relationships $\bar{\mathbf{c}} = \mathbf{c}$, $\bar{\mathbf{s}} = \mathbf{s}$, and $\bar{\mathbf{d}} = \mathbf{d}$ still hold at the continuum limit.

[1] Guevara Vasquez, F., Milton, G. W., Onofrei, D. & Seppecher, P. Transformation elastodynamics and active exterior acoustic cloaking. In *Acoustic Metamaterials*, 289–318 (Springer, 2013).
