# Supplementary material for: Grounded reconfigurable metamaterials with customized mapping-invariant behavior
Source: Nat Commun. 2026 May 19;17:6572. doi: 10.1038/s41467-026-73240-8 (PMC13381600; doi:10.1038/s41467-026-73240-8)
Supplement: Supplementary file 2 — Transparent Peer Review File [file 41467_2026_73240_MOESM2_ESM.pdf]

nature portfolio

Peer Review File

Grounded reconfigurable metamaterials with customized mapping-invariant behavior

Corresponding Author: Dr Yangyang Chen

This file contains all reviewer reports in order by version, followed by all author rebuttals in order by version.

Version 0:

Reviewer comments:

Reviewer #1

(Remarks to the Author)
In this work, the authors present a model based on a triangular tessellation shape with reconfigurable hinges which can undergo complex shape morphing behaviour. Although the work appears to be scientifically sound, the novelty of the work is not clear to me from the presentation of the work, particularly, the "grounded" aspect. There is also significant emphasis on "non-standard" deformation modes, yet it is unclear exactly what this entails. In my opinion, the authors need to clarify more clearly exactly what is the novelty and applicability of their work. Detailed comments are listed below:

a) The images in the supplementary information show that the metamaterials in question are attached by rods to a flat surface while also constrained from the sides. Given these strict constraining requirements, the question arises whether this concept can be easily replicated at smaller scales such as the microlevel and also how the concept can be reasonably implemented in real-life applications.

b) There is no information on the resultant mechanical properties of these metamaterial systems after loading.

c) Can reconfiguration be attained through other methods apart from hinges? Because as assembled systems, the applicability of these metamaterials is limited at smaller scales due to fabrication methodology limitations.

d) The novelty of the concept must be explained more clearly.

Reviewer #2

(Remarks to the Author)
Key results
The manuscript proposes concept of grounded mechanical metamaterials, which is typically seen in electromagnetics but not in mechanical/dynamic metamaterials. To the best of the knowledge of this reviewer, such a concept is new within the mechanical/dynamic metamaterials and offers additional possibilities for embedding new types of functionalities into metamaterials.

Validity
The research seems valid from the theoretical and methodological perspective. The chosen approach is reasonable, and derivations of the main concepts are described. These mostly involve static equilibrium of kinematic rigid mechanisms with embedded torsional springs. The developed theory and concepts are validated also experimentally, further confirming obtained results. Experiments seem to be trustable.

Significance
The concept of grounding is interesting, to the best of the knowledge of this reviewer novel, and offers possibilities for embedding new interesting functionalities within metamaterials. However, it also poses limitations on the type and design of metamaterials, since grounding might not be feasible/easily implemented for full 3D metamaterials, but might be easier for planar structures.

Data and methodology
Theoretical and experimental methodology seems sound (cf. also Validity above). Quality of the data is good, presentation might offer some space for improvement (cf. Suggested improvements below).

Analytical approach
Adequate, involving static equilibrium of kinematic rigid mechanisms with embedded torsional springs and external supports. Some room for improvement is present, as it is not always clear to the reviewer the kinematic setup of internal and external constraints (cf. Suggested improvements below). Approach towards experimental validation seems sound, although that lies at the edge of the expertise of this reviewer.

Suggested improvements
The main text of the manuscript might benefit from the following improvements (listed chronologically):
1. Introduction: “their intended performance after substantial deformation.”. I agree with the statement, but later, GMM shows reconfiguration rather than morphing/deformation. See also caption of Fig. 2 where authors themselves use configuration: “The metamaterial is configured…”.
2. Fig. 1 and elsewhere: it would be beneficial to clearly indicate graphically fixed, free, and elastic hinges (e.g., in color, or some other marking). Pictures from CAD are nice, but not really representative of the mechanics/kinematic assumptions adopted within the system.
3. General comment: morphing is in my opinion not a suitable choice of wording, as in my understanding configuration needs to change (cf., e.g., Figs 2a-2c, where adjustable levers are clearly in different configurations). The material does not continuously/elastically deform from one configuration to other, as far as I understand.
4. General comment: authors consider in the system two kinds of springs: (real) springs (Fig. 1a) and effective lever springs (Fig. 1b). In the text both are interchangeably referred to as springs. It would help readability removing this ambiguity by introducing a fixed naming convention for each of the cathegories of the springs and strictly sticking to it throughout the text. This could be, for instance, springs and lever springs.
5. General comment: the authors call the mechanism Willis coupling. In my understanding, it is simply linkage with another spring, fixed to the external world, hence displacements couple with forces, unlike, e.g., momentum density and strain, two distinct quantities. Is Willis coupling really substantiated here? Deeper clarification would be beneficial.
6. Page 3: “Clearly, when the distance between nodes A and C changes…”. BA can also rotate around B, thus changing AC distance without any torque. Please amend.
7. Eq. (3): deserves reference to supporting material where expressions are explained in more detail.
8. General methodology comment: Currently the explanation is devised bottom-up through deduction steps starting from a simple mechanism by generalization, and is not fully straightforward to follow (especially rotation constraints are not fully clear, cf. Comment 2 above). All intermediate turns might be clouding unnecessarily the main working mechanism. Consider adding more specific details to improve readability or going top-down, i.e., introducing the ultimate mechanism and deductively reveal its functionality.
9. General comment: authors refer to restrained rotation of rigid masses. Those are, in my understanding, restrained due to two possible mechanisms: by the elastic lever mechanisms representing the springs (Fig. 1d), and by pinning/grounding them through magnets (Fig. 1e). If this is correct, similarly to Comment 4 above, consider specific naming convention for the two types of rotational restraints for better readability and using it consistently throughout the text.
10. Fig. 2h: consider enlarging inset of point mass numbering.
11. Figs. 2g-2h and elsewhere: horizontal axis’ caption could be renamed (e.g., rigid mass ID/number). In my impression improving naming consistency throughout the entire text would improve readability significantly (points vs. masses, two kinds of springs, two kinds of rotation restraints, morphing/reconfiguration, all combined together was at times difficult for me to follow the main developments and it was not clear what or which mechanism authors exactly mean).
12. Page 5: effective properties of mechanical metamterials are often discussed in the literature. A discussion on, e.g., effective stiffness/anisotropy of GMMs as a function of their configuration would be interesting addition.
13. Fig. 3f and related discussion in the text: it is not clear to me how the system is deformed. Are only the two points 3-4 displaced horizontally in the -x_1 direction? Additional explanation would be helpful.
14. Discussion: “morphing-invariant” might be an overstatement in my opinion, cf. Comments 1 and 3 above.

Supplementary material might benefit from the following changes:
15. Fig. S1: black font for C and D is poorly visible on the dark background.
16. Page 2: “Universal joints and a rigid bar and is attached to the ground”. See Comment 9 above; is the rotation restrained in a stiff manner, or is there elasticity in the rotational constraint?
17. S4: numerical simulations would benefit from more detailed information (mesh, type of simulation, solvers, etc.). This would certainly substantiate the space needed.
18. Figs. S6, S7, S9, S11, S12, S13, S14, S15: missing labels a., b., etc.

Clarity and context
The main message of the contribution is currently not fully clear to me, although morphing-invariant metamaterials is stands out. The issue I have might be more related to small inconsistencies and gaps in the precise methodology and design, which might improve with the suggestions listed above. The proposed concept relates well to work within mechanical metamaterials.

References
The body of existing literature is sampled well, with no particular suggestions from this reviewer.

Your expertise

Experimental validation is at the edge of the expertise of this reviewer.

Version 1:

Reviewer comments:

Reviewer #1

(Remarks to the Author)
The authors have improved the manuscript significantly and it can now be accepted for publication

Reviewer #2

(Remarks to the Author)
The authors have sufficiently addressed my comments, hence it is my pleasure to recommend the manuscript for acceptance.

Open Access This Peer Review File is licensed under a Creative Commons Attribution 4.0 International License, which permits use, sharing, adaptation, distribution and reproduction in any medium or format, as long as you give appropriate credit to the original author(s) and the source, provide a link to the Creative Commons license, and indicate if changes were made.
In cases where reviewers are anonymous, credit should be given to 'Anonymous Referee' and the source.
The images or other third party material in this Peer Review File are included in the article’s Creative Commons license, unless indicated otherwise in a credit line to the material. If material is not included in the article’s Creative Commons license and your intended use is not permitted by statutory regulation or exceeds the permitted use, you will need to obtain permission directly from the copyright holder.
To view a copy of this license, visit https://creativecommons.org/licenses/by/4.0/

Response to Reviewers' Comments

The authors would like to take this opportunity to thank the reviewers for their insight and suggestions. We have responded to the comments below, and the paper has been modified accordingly. All the revised and added parts have been highlighted in blue in the revised manuscript.

Reviewer 1

> In this work, the authors present a model based on a triangular tessellation shape with reconfigurable hinges which can undergo complex shape morphing behaviour. Although the work appears to be scientifically sound, the novelty of the work is not clear to me from the presentation of the work, particularly, the "grounded" aspect. There is also significant emphasis on "non-standard" deformation modes, yet it is unclear exactly what this entails. In my opinion, the authors need to clarify more clearly exactly what is the novelty and applicability of their work.

Reply: We thank the reviewer for his/her constructive feedback and the observation that the work is scientifically sound. We appreciate the opportunity to clarify the novelty of the utility of the transformation method, the "grounded" architecture, and the realization of static Willis coupling. Beyond that, we also illustrate the nature of "non-standard" deformation modes and the applicability of the grounded metamaterial. We have revised the manuscript to emphasize these points, as summarized below.

- 1. Novelty—leveraging transformation method to design grounded metamaterials with customized mapping-invariant displacement fields

  The primary novelty lies in expanding the utility of the transformation method. While coordinate transformation has traditionally been used for wave control (e.g., acoustic cloaking or elastic lenses), we have unlocked a new usage for designing materials to achieve customized mapping-invariant static displacement fields. In particular, we describe the reconfiguration of the positions of microstructures associated with material shape changes by the geometric mapping of coordinate transformation and correlate the customized static displacement field to the displacement gauge implemented into the transformation. This way, we identify an inverse design guideline for metamaterials with desired mapping-invariant displacement fields. Unlike transformation optics or acoustics, transformation elasticity in solids requires the material to be polar and/or exhibit Willis coupling to satisfy the transformation-invariance. The grounded architecture is the physical mechanism that enables this by allowing the material to interact with an external reference frame to host body forces and body torques otherwise impossible in free-standing materials.

– 1.1. Grounded mass—origin of body torque

The "grounded" aspect is not a peripheral mounting choice, but a fundamental theoretical requirement for facilitating transformation elasticity in solids under quasi-static assumptions. In traditional free-standing metamaterials, substantial shape changing usually causes microstructure rearrangement, which necessarily alters their mechanical responses because internal forces must adapt to the new microstructure and geometric configuration. Our grounded metamaterials (GMMs) are the first to achieve configuration-mapping-invariance, where the displacement response remains identical regardless of whether the metamaterial is a rectangle, a parallelogram, or an arbitrary shape with any possible microstructure arrangements. Physically, internal forces in free-standing lattices must be concurrent for equilibrium. Once reconfigured, directions and magnitudes of these forces must change to satisfy equilibrium conditions, so as the displacement response must also alter. Only a grounded material can keep those forces unchanged by providing necessary reaction body torques to balance the non-concurrent internal forces arose from reconfiguration, achieving mapping-invariance. Without the ground connection to suppress mass rotation (achieved via our universal joint system), the metamaterial would simply rotate locally and vary in performance upon reconfiguration. Thus, the body torques in the grounded metamaterial are the key for generating the mapping-invariant displacement field. This interaction allows the metamaterial to break the minor symmetries of the stiffness tensor and display polarity, enabling behaviors physically forbidden in free-standing structures.

– 1.2. Static Willis spring—origin of body force

Another significant contribution of this work is the mechanical realization of static Willis coupling. Willis coupling has been studied for decades in the dynamic regime, but it typically vanishes as frequency approaches zero ($\omega \to 0$). Our four-bar linkage is the first spring design to display significant Willis coupling in the static limit. Specifically, the kinematics of our four-bar linkage naturally yield internal and grounded forces, $\mathbf{f}^d_{AC}$ and $\mathbf{f}^d_{E}$, that automatically satisfy the coupled constitutive requirements derived from transformation equations. This ensures the metamaterial hosts desired displacement fields without needing active control systems. The static Willis springs are the core for achieving any user-defined displacement fields in GMMs. Physically, this behavior can be attributed to the grounded connection in the Willis spring that provides a carefully tailored body force applied on the mass to manipulate the displacement field in a desired way. It is also worth mentioning that the displacement field of the Willis metamaterial is still invariant to microstructure and geometric reconfiguration as long as the Willis springs and their connections remain the same, since the elastic energy of each of the Willis springs leaves unchanged, and the net torques due to reconfiguration are balanced by reaction torques from rotation-suppressed masses.

- 2. Clarifying non-standard deformation modes

In the manuscript, "non-standard" deformation modes refer to displacement fields that are physically forbidden in classical, free-standing elastic solids. These behaviors are enabled by the grounded masses and the implementation of static Willis springs. The specific examples demonstrated in the paper and supplementary material include: (i) Asymmetric tension without bending: In standard materials, asymmetric tensile loading (eccentric loads) inevitably

induces a bending moment. In the GMM, the Willis springs carefully manage the asymmetric displacement field through their kinematics that effectively cancel this moment, allowing the material to remain perfectly planar while hosting an asymmetric internal tension field. (ii) Tension-induced buckling (or bulging): Buckling is typically a failure mode associated with compression. This research demonstrates a mode where horizontal tension induces a programmed vertical "bulge" or upward bend. This is achieved through a displacement gauge that creates a stable, off-diagonal cross-coupling between horizontal and vertical displacement components. (iii) Tailored zero-energy modes: The GMM can be programmed such that complex deformations—for example, a combination of rigid-body translation and shear—cost zero elastic energy. In classical mechanics, shear deformation would require work to deform the internal springs, and only rigid-body translation and rotation cost zero elastic energy, but in a GMM, this combination can be treated as "customized" zero-mode motions. (iv) Independent curvature control: The material allows for the independent control of bending curvatures for different longitudinal fibers. For example, under a single bending load, the top, middle, and bottom layers can be programmed to exhibit entirely different curvatures or non-planar vertical cross-sections. (v) Nonstandard rigid-body rotation: Demonstrated in the supplementary material, this involves rotational displacement fields that depart from classical rigid-body kinematics through the application of a displacement gauge. These modes are physically realized using Willis springs with pinned ground supports and rotation-suppressed masses, which allow the material to host the necessary body forces and body torques to induce these unconventional states.

- 3. Applicability

  While the prototype uses magnets and rods for concept demonstrations, the theoretical framework proposed in the manuscript is scalable and offers substantial impact across multiple engineering domains. For example, in aerospace and soft robotics, GMMs enable morphing structures and sensing "skins" that maintain calibrated performance regardless of their posture or configuration. Furthermore, integrating speakers or other active components on the top surfaces of the mass nodes in GMMs allows for tunable acoustic and electromagnetic manipulation through customized mechanical deformation, enabling reconfigurable phase-modulating devices. Finally, the GMMs can be applied to support telescope mirrors to allow reconfiguration and, at the same time, to maintain position accuracy. They can also be employed for optimizing mirror focus through customized mechanical deformation in photothermal power plants. We have illustrated more details regarding the applicability in Response 1.1.

> **1.1** The images in the supplementary information show that the metamaterials in question are attached by rods to a flat surface while also constrained from the sides. Given these strict constraining requirements, the question arises whether this concept can be easily replicated at smaller scales such as the microlevel and also how the concept can be reasonably implemented in real-life applications.

Reply: We appreciate the reviewer's insightful comments regarding the practical implementation of our grounded metamaterials. We have addressed these points in the revised manuscript and provided the following clarifications.

It should be noted that the rod-and-magnet assembly demonstrated in the manuscript is a centimeter-scale laboratory prototype. However, the theoretical requirements of rotation suppression and static Willis coupling derived from the transformation method are scale-independent and can be replicated at smaller scales. In particular, at millimeter- or even micrometer-scales, the traditional pin-jointed hinges can be replaced by monolithic compliant mechanisms, such as diamond-shaped bars with extremely small tip connections. By leveraging the high aspect ratio of these flexures, the transmission of moments can be effectively ignored, mimicking the behavior of a physical pin. Further, the requirement to suppress mass rotation while allowing translation and the ground connections in Willis springs can be achieved without physical rods. In millimeter- or micrometer-scale systems, electrostatic or magnetic grounding can be utilized to provide the necessary body torques and reaction forces directly to the masses or the ground nodes of the Willis springs. These forces interact with an underlying substrate (serving as the "ground"), enabling the material to maintain its customized response without bulky mechanical constraints.

Beyond the structural benefits mentioned in the manuscript, the GMM offers a unique platform for multifunctional engineering integration in real-life applications. (i) Acoustic wave field control: Integrating an arrays of speakers on the top surface of the grounded masses allows for the active manipulation of sound waves and phonons. For this application, GMMs can function as tunable sound generators by leveraging their customized deformation to manipulate the positions of the speakers in demand for different desired sound fields. (ii) Electromagnetic manipulation: Similar to sound manipulation, GMMs provide a physical platform where the electromagnetic wave field can be mechanically reconfigurable, enabling antennas that can be simply tuned using programmed mechanical deformations. (iii) Mechanical screens and light modulation: By adjusting the spacing and orientation of elements, GMMs can act as "mechanical screens" for light reflection. They can function as metagratings for anomalous reflection or phase modulation, providing a highly efficient reconfigurable platform for next-generation flat optical devices. (iv) Telescope mirror support: High-precision telescopes require mirror supports that maintain surface form accuracy. GMMs can be suited for this, as they can provide the high stiffness and grounded reaction torques necessary to compensate for gravity-induced elastic deformation across all pointing angles. At the same time, GMMs also allow reconfiguration and folding necessary to the transportation of telescope mirrors. (v) Photothermal systems: In photothermal power plants, large arrays of sun-tracking mirrors focus energy onto thermal receivers. GMM-based control can optimize mirror support structures to handle structural reconfigurations or thermal variations without losing the precision required for energy capture.

Finally, we would like to clarify that the side constraints observed in the Supplementary Information are part of the experimental boundary conditions required to apply controlled tension and bending for verification, rather than a functional requirement of the metamaterial itself. Once the GMM is attached to its primary reference frame (the "ground"), the material is mechanically self-contained.

> **1.2** There is no information on the resultant mechanical properties of these metamaterial systems after loading.

Reply: We appreciate the reviewer's comment on clarification of the resultant mechanical properties of our grounded metamaterials transformed with different geometric mapping. We first revisit the continuum transformation elasticity and then perform homogenization for the grounded metamaterials derived from discrete transformation with different geometric mapping. Finally, we compare

the effective elastic properties with those from continuum transformation. These discussions have been added into SI.

To formulate the continuum transformation, we first consider a virtual elastic solid occupied in $\mathbf{X}$ and assume that the virtual solid obeys Hooke's law. The virtual solid deforms with the displacement field $\mathbf{U(X)}$ in response to a given set of external loads or constraints. Our goal is to calculate the material properties of the transformed solid to realize the desired displacement field $\mathbf{u(x)}$ in a transformed coordinate $\mathbf{x}$. To facilitate the coordinate transformation, it is useful to write the elastic energy density of the virtual solid as

$$\mathcal{E} = \frac{1}{2} C_{ijkl} \frac{\partial U_j}{\partial X_i} \frac{\partial U_l}{\partial X_k}, \tag{1}$$

where C_{ijkl} denotes the elasticity tensor of the virtual solid, and the stress tensor reads $\Sigma_{ij} = C_{ijkl} \frac{\partial U_l}{\partial X_k}$. Note that we only consider linear deformations and Cauchy elasticity. To achieve the desired displacement field $\mathbf{u(x)}$ in the physical solid, we introduce a displacement gauge $\mathbf{B(X)}$ defined as [1]

$$\mathbf{u(x)} = \left(\mathbf{B(X)}^T\right)^{-1} \mathbf{U(X)}, \tag{2}$$

where $\mathbf{B(X)}$ is an invertible matrix. The spatial derivative of the virtual displacement field then read

$$\frac{\partial U_j}{\partial x_i} = B_{pj} \frac{\partial u_p}{\partial x_i} + \frac{\partial X_q}{\partial x_i} \frac{\partial B_{pj}}{\partial X_q} u_p. \tag{3}$$

Implementing coordinate transformation by substituting Eq. (3) into Eq. (1), we obtain the elastic energy density in $\mathbf{x}$ as

$$e = \frac{1}{2} \left(\frac{\partial u_j}{\partial x_i} \sigma_{ij} + u_k f_k \right), \tag{4}$$

$$\sigma_{ij} = c_{ijkl} \frac{\partial u_l}{\partial x_k} + s_{ijk} u_k, \tag{5}$$

$$f_k = s_{ijk} \frac{\partial u_j}{\partial x_i} + d_{kl} u_l, \tag{6}$$

where $c_{ijkl} = \frac{1}{J} C_{pqst} \frac{\partial x_i}{\partial X_p} B_{jq} \frac{\partial x_k}{\partial X_s} B_{lt}$, $s_{ijk} = \frac{1}{J} C_{pqst} \frac{\partial x_i}{\partial X_p} B_{jq} \frac{\partial B_{kt}}{\partial X_s}$, $d_{kl} = \frac{1}{J} C_{pqst} \frac{\partial B_{kq}}{\partial X_p} \frac{\partial B_{lt}}{\partial X_s}$ with $J = \det(\frac{\partial x_i}{\partial X_j})$, and e, σ_{ij}, and f_k denote the elastic energy density, stress, and body force of the transformed solid, respectively. Clearly, the elastic energy density of the transformed solid e no longer keeps its original form after the transformation. Instead, it has two parts: one comes from the strain energy density; the other arises due to the work done by the body force, a new term caused by the transformation. Further, the stress-strain relationship of the transformed solid is no longer Hooke's law, as noted by inspection of Eq. (5) and the definition of s_{ijk}, which depends on the transformation. Equation (5) shows that stresses in the transformed solid depend on both the displacement gradients and the displacement itself through a elastostatic Willis coupling tensor s_{ijk}. In a similar

manner, body forces are determined by not only the displacement but the displacement gradients through the same Willis coupling tensor s_{ijk}. Secondly, the elasticity tensor of the transformed solid loses minor symmetry, e.g. $c_{ijkl} \neq c_{ijlk} \neq c_{jilk}$, leading to asymmetric stresses, a response characterizing elastic polarity. Once the two properties are satisfied in a solid material, the desired displacement behavior will be guaranteed. The observations here are identical with that derived from the discrete transformation shown in the main text.
Next, we homogenize the GMM design in the main text and calculate its effective elasticity tensors. The potential energy per unit area is calculated under a macroscopic displacement gradient $\mathbf{e}$ and a uniform displacement $\mathbf{u}$ over a unit cell of the GMM. For the triangular lattice demonstrated in the examples, we decompose the potential energy density $\bar{\varepsilon}$ into three parts caused by the three springs $\{i,j\}$, $\{i,k\}$, and $\{j,k\}$ in the unit cell. The potential energy density then reads

$$\bar{\varepsilon} = \bar{\varepsilon}_{ij} + \bar{\varepsilon}_{ik} + \bar{\varepsilon}_{jk}, \tag{7}$$

where

$$\bar{\varepsilon}_{ij} = \frac{k_{ij}^d}{2A}\left(\mathbf{e}\mathbf{r}_{ij}\mathbf{n}_{ij}^d\right)^2 + \frac{\hat{k}_{ij}^d}{A}\left(\mathbf{e}\mathbf{r}_{ij}\mathbf{n}_{ij}^d\right)\left(\mathbf{u}\mathbf{t}_{ij}^d\right) + \frac{\tilde{k}_{ij}^d}{2A}\left(\mathbf{u}\mathbf{t}_{ij}^d\right)^2, \tag{8}$$

$$\mathbf{r}_{ij} = \mathbf{x}_i - \mathbf{x}_j, \tag{9}$$

and A is the area of the unit cell. The effective elasticity tensors of the GMM can be derived by

$$\bar{c}_{ijkl} = \frac{\partial^2 \bar{\varepsilon}}{\partial e_{ij} \partial e_{kl}}, \tag{10}$$

$$\bar{s}_{ijk} = \frac{\partial^2 \bar{\varepsilon}}{\partial e_{ij} \partial u_k}, \tag{11}$$

$$\bar{d}_{ij} = \frac{\partial^2 \bar{\varepsilon}}{\partial u_i \partial u_j}. \tag{12}$$

Calculating Eqs. (10-12) at the continuum limit, the effective elasticity tensors are equal to those derived from continuum transformation, $\bar{c}_{ijkl} = c_{ijkl}$, $\bar{s}_{ijk} = s_{ijk}$, and $\bar{d}_{ij} = d_{ij}$.
Next, we validate these relationships through two illustrative examples by calculating effective elastic properties using continuum transformation elasticity and performing homogenization for the grounded metamaterials. We assume a free-standing triangular lattice in the virtual space (as shown in the main text) with uniform spring constant K_0. Its effective elasticity tensor reads

$$\mathbf{C} = \begin{bmatrix} \lambda + 2\mu & \lambda & 0 & 0 \\ \lambda & \lambda + 2\mu & 0 & 0 \\ 0 & 0 & \mu & \mu \\ 0 & 0 & \mu & \mu \end{bmatrix}, \tag{13}$$

where $\lambda = \mu = \frac{\sqrt{3}K_0}{4}$.
Example I:

We select the displacement gauge $\mathbf{B} = \begin{bmatrix} 1 & -b(X_1) \\ 0 & 1 \end{bmatrix}$ and $\mathbf{x} = \mathbf{X}$ corresponding to the example demonstrated in Fig. 4a. Implementing this displacement gauge and coordinate mapping into Eqs. (4-6), transformed elasticity tensors derived from continuum transformation can be written as

$$\mathbf{c} = \mu \begin{bmatrix} 3+b^2 & 1 & -b & -2b \\ 1 & 3 & 0 & -3b \\ -b & 0 & 1 & 1 \\ -2b & -3b & 1 & 1+3b^2 \end{bmatrix}, \tag{14}$$

$$\mathbf{s} = \mu \begin{bmatrix} b\frac{\partial b}{\partial X_1} & 0 \\ 0 & 0 \\ -\frac{\partial b}{\partial X_1} & 0 \\ -\frac{\partial b}{\partial X_1} & 0 \end{bmatrix}, \tag{15}$$

$$\mathbf{d} = \mu \begin{bmatrix} (\frac{\partial b}{\partial X_1})^2 & 0 \\ 0 & 0 \end{bmatrix}. \tag{16}$$

Next, we perform homogenization for the grounded metamaterial shown in Fig. 4a based on Eqs. (7-12), the effective elasticity tensors are attained as

$$\bar{\mathbf{c}} = \frac{\sqrt{3}K_0}{4} \begin{bmatrix} 3+b_j^2 & 1 & -b_j & -2b_j \\ 1 & 3 & 0 & -3b_j \\ -b_j & 0 & 1 & 1 \\ -2b_j & -3b_j & 1 & 1+3b_j^2 \end{bmatrix}, \tag{17}$$

$$\bar{\mathbf{s}} = \frac{\sqrt{3}K_0}{4} \begin{bmatrix} \frac{2(b_j-b_k)}{a_0}b_j & 0 \\ 0 & 0 \\ -\frac{2(b_j-b_k)}{a_0} & 0 \\ -\frac{2(b_j-b_k)}{a_0} & 0 \end{bmatrix}, \tag{18}$$

$$\bar{\mathbf{d}} = \frac{\sqrt{3}K_0}{4} \begin{bmatrix} \frac{4(b_j-b_k)^2}{a_0^2} & 0 \\ 0 & 0 \end{bmatrix}, \tag{19}$$

where b_j denotes $b(\mathbf{X}_j)$. Comparing Eqs. (17-19) with Eqs. (14-16), it can be clearly seen that $\bar{\mathbf{c}} = \mathbf{c}$, $\bar{\mathbf{s}} = \mathbf{s}$, and $\bar{\mathbf{d}} = \mathbf{d}$ at the continuum limit.
Example II:
In the second example, we consider the parallelogram mapping corresponding to the grounded metamaterial in Fig. 4e, where $\nabla_X \mathbf{x} = \begin{bmatrix} 1 & 0 \\ \gamma & 1 \end{bmatrix}$, γ is a constant. Again, the transformed elasticity tensors derived from continuum transformation can be written as

$$\mathbf{c} = \mu \begin{bmatrix} 3+b^2 & 1-\gamma b & -b & 3\gamma-2b+\gamma b^2 \\ 1-\gamma b & 3+\gamma^2 & \gamma & 2\gamma-3b-\gamma^2 b \\ -b & \gamma & 1 & 1-\gamma b \\ 3\gamma-2b+\gamma b^2 & 2\gamma-3b-\gamma^2 b & 1-\gamma b & 1+3\gamma^2-4\gamma b+3b^2+\gamma^2 b^2 \end{bmatrix}, \tag{20}$$

$$\mathbf{s} = \mu \begin{bmatrix} b\frac{\partial b}{\partial X_1} & 0 \\ -\gamma\frac{\partial b}{\partial X_1} & 0 \\ -\frac{\partial b}{\partial X_1} & 0 \\ (\gamma b - 1)\frac{\partial b}{\partial X_1} & 0 \end{bmatrix}, \tag{21}$$

$$\mathbf{d} = \mu \begin{bmatrix} (\frac{\partial b}{\partial X_1})^2 & 0 \\ 0 & 0 \end{bmatrix}. \tag{22}$$

The homogenized elasticity tensors read

$$\bar{\mathbf{c}} = \frac{\sqrt{3}K_0}{4} \begin{bmatrix} 3 + b_j^2 & 1 - \gamma b_j & -b_j & 3\gamma - 2b_j + \gamma b_j^2 \\ 1 - \gamma b_j & 3 + \gamma^2 & \gamma & 2\gamma - 3b_j - \gamma^2 b_j \\ -b_j & \gamma & 1 & 1 - \gamma b_j \\ 3\gamma - 2b_j + \gamma b_j^2 & 2\gamma - 3b_j - \gamma^2 b_j & 1 - \gamma b_j & 1 + 3\gamma^2 - 4\gamma b_j + 3b_j^2 + \gamma^2 b_j^2 \end{bmatrix}, \tag{23}$$

$$\bar{\mathbf{s}} = \frac{\sqrt{3}K_0}{4} \begin{bmatrix} \frac{2(b_j - b_k)}{a_0} b_j & 0 \\ -\frac{2(b_j - b_k)}{a_0}\gamma & 0 \\ -\frac{2(b_j - b_k)}{a_0} & 0 \\ \frac{2(b_j - b_k)}{a_0}(\gamma b_j - 1) & 0 \end{bmatrix}, \tag{24}$$

$$\bar{\mathbf{d}} = \frac{\sqrt{3}K_0}{4} \begin{bmatrix} \frac{4(b_j - b_k)^2}{a_0^2} & 0 \\ 0 & 0 \end{bmatrix}. \tag{25}$$

Comparing Eqs. (23-25) with Eqs. (20-22), the relationships $\bar{\mathbf{c}} = \mathbf{c}$, $\bar{\mathbf{s}} = \mathbf{s}$, and $\bar{\mathbf{d}} = \mathbf{d}$ still hold at the continuum limit.

1.3 Can reconfiguration be attained through other methods apart from hinges? Because as assembled systems, the applicability of these metamaterials is limited at smaller scales due to fabrication methodology limitations.

Reply: Thanks for the reviewer's foresight regarding the fabrication challenges at smaller scales. To fully address this, it is helpful to first distinguish between the two mechanical roles played by hinges and reconfiguration mechanisms in our current laboratory prototypes. Hinges (or pin-joints) are implemented to connect the masses to the bars of Willis springs, allowing the necessary kinematic rotation. They only transmit forces, forbidding any moment transmission. Reconfiguration mechanisms in our prototype are realized via grooved bars and screws to allow manually adjusting positions of masses and Willis springs (e.g. from Config. I to Config. II in Fig. 2).
We agree that the assembled approach used for our laboratory prototype is not suitable for small-scale applications. However, as pointed in Response 1.1, the design principles of GMMs are still valid at smaller scales. To assist manufacturing, the hinges can be replaced by flexural or compliant joints (i.e. using fabric or diamond-shaped bars with small tip connections). Further, electrostatic or magnetic grounding can be utilized to provide the necessary body torques and reaction forces.

Thus, the entire GMM can be fabricated via monolithic fabrication processes, such as multi-material 3D printing. This fabrication method would eliminate the need for assembly.

It should be noticed that while our current prototype uses manual screws for reconfiguration, this is merely a demonstration method. In advanced applications, the grooved bars and screws can be replaced by circuit-controlled devices, such as electromagnetic linear stage or pneumatic/hydraulic actuators. This would allow the GMM to undergo active and autonomous reconfiguration in response to electrical signals, thereby removing the need for manual adjustment.

1.4 The novelty of the concept must be explained more clearly.

Reply: We thank the reviewer for pointing out the need to clarify the novelty. We agree that explicitly summarizing the unique contributions of this work strengthens the manuscript. In the revised version, we have added a dedicated summary in both the Introduction and Discussion sections to distill the core novelty as shown below.

This work unlocks a new application for the transformation method, expanding its utility from wave manipulation to the precise design of materials with mapping-invariant and customized static displacement fields. By introducing a grounded architecture, we decouple mechanical performance from geometric configuration, overcoming the shape-performance trade-off that has historically limited the reliability of reconfigurable systems. The grounded aspect is shown to be a fundamental physical requirement for transformation elasticity in solids, providing the necessary reaction body torques to balance non-concurrent internal forces that arise during substantial reconfiguration.

A core technical breakthrough is the mechanical realization of the first static Willis spring—a four-bar linkage that achieves desired Willis coupling in the static limit ($\omega \to 0$), a regime where such coupling traditionally vanishes. The kinematics of this spring design automatically satisfy the complex material parameter requirements of transformation elasticity, enabling non-standard deformation modes that are forbidden in classical elasticity, such as asymmetric tension without bending and tension-induced buckling. This scalable framework offers transformative potential for high-precision adaptive systems, including aerospace morphing structures and precision mirror supports for telescopes and solar energy harvesting.

Reviewer 2

Key results
The manuscript proposes concept of grounded mechanical metamaterials, which is typically seen in electromagnetics but not in mechanical/dynamic metamaterials. To the best of the knowledge of this reviewer, such a concept is new within the mechanical/dynamic metamaterials and offers additional possibilities for embedding new types of functionalities into metamaterials.
Validity
The research seems valid from the theoretical and methodological perspective. The chosen approach is reasonable, and derivations of the main concepts are described. These mostly involve static equilibrium of kinematic rigid mechanisms with embedded torsional springs. The developed theory and concepts are validated also experimentally, further confirming obtained results. Experiments seem to be trustable.
Significance
The concept of grounding is interesting, to the best of the knowledge of this reviewer novel, and offers possibilities for embedding new interesting functionalities within metamaterials. However, it also poses limitations on the type and design of metamaterials, since grounding might not be feasible/easily implemented for full 3D metamaterials, but might be easier for planar structures.
Data and methodology
Theoretical and experimental methodology seems sound (cf. also Validity above). Quality of the data is good, presentation might offer some space for improvement (cf. Suggested improvements below).
Analytical approach
Adequate, involving static equilibrium of kinematic rigid mechanisms with embedded torsional springs and external supports. Some room for improvement is present, as it is not always clear to the reviewer the kinematic setup of internal and external constraints (cf. Suggested improvements below). Approach towards experimental validation seems sound, although that lies at the edge of the expertise of this reviewer.

Reply: We would like to express our sincere gratitude to the reviewer for his/her positive and detailed assessment of our manuscript. We thank the reviewer that he/she found our concept of grounded metamaterials both novel and scientifically valid, noting its potential for embedding new types of functionalities within the field. We particularly value the reviewer's confirmation of our experimental results as trustable and his/her constructive feedback on the presentation of our kinematic setups and constraint implementations. We have carefully considered all suggested improvements to enhance the clarity and impact of the work.

2.1 Introduction: "their intended performance after substantial deformation.". I agree with the statement, but later, GMM shows reconfiguration rather than morphing/deformation. See also caption of Fig. 2 where authors themselves use configuration: "The metamaterial is configured...".

Reply: We thank the reviewer for this insightful observation regarding our terminology. We agree that a clear distinction should be made between the reconfiguration of the metamaterial's geometry (the discrete process of mapping/repositioning the nodes) and the deformation it undergoes in response to external loads (the continuous displacement fields with respect to the reference geometry of one configuration). As suggested, we have revised the manuscript to ensure consistency.

In the Introduction, the phrase "substantial deformation" has been updated to "microstructure and geometric reconfigurations" to accurately describe the process by which the material changes its shape (or the reference geometry). We have also replaced "morphing-invariant" with "mapping-invariant" in the title and throughout the text to emphasize that performance is decoupled from the geometric mapping process rather than from the load-induced deformation itself. These changes clarify that our framework ensures intended performance is preserved even after the material's architecture is radically reconfigured.

Key changes have been made in the revised manuscript to highlight this distinction:

Title: Changed to "Grounded reconfigurable metamaterials with customized mapping-invariant behavior".

Introduction: Revised the sentence to: "However, mechanical metamaterials often fail to preserve their intended performance after microstructure reconfigurations to meet the desired shape transformations".

Figure Captions: Consistently used "configured" or "configurations" (e.g., Configuration I, II, and III) instead of "deformation" when referring to the shape of the lattice during testing.

> **2.2** Fig. 1 and elsewhere: it would be beneficial to clearly indicate graphically fixed, free, and elastic hinges (e.g., in color, or some other marking). Pictures from CAD are nice, but not really representative of the mechanics/kinematic assumptions adopted within the system.

Reply: We thank the reviewer for this helpful suggestion. We agree that while CAD renderings provide a realistic view of the physical assembly, they may not fully capture the underlying kinematic assumptions. To address this, we have extensively reorganized and enriched the illustrations in the revised manuscript.

In the revised Fig. 1 and elsewhere, we have adopted standard mechanical diagrams to clearly show the system's topology and constraints:

1. Pin connections (free hinges) are now explicitly represented by open circles, indicating free rotation.
2. Grounded constraints of Willis springs are clearly marked with standard grounding symbols.
3. Elastic/Willis elements are schematically highlighted. Specifically, we use double-headed curved arrows to represent the Willis coupling effect, which is physically realized by the elastic cylinder connecting the bars in our proposed four-bar linkage mechanism. We also provide kinematic representations composed of rigid bars and torsional springs to clearly show mechanics assumptions.

These schematic enhancements ensure that the mechanics and kinematic assumptions are unambiguously conveyed to the reader.

2.3 General comment: morphing is in my opinion not a suitable choice of wording, as in my understanding configuration needs to change (cf. e.g. Figs 2a-2c, where adjustable levers are clearly in different configurations). The material does not continuously/elastically deform from one configuration to other, as far as I understand.

Reply: We appreciate the reviewer's precision regarding the wording. We agree that morphing often implies a continuous or elastic transition between states, whereas our material achieves its shape changes through a discrete process of reconfiguration by loosening screws, repositioning the starburst mass arms, which can be better described by re-mapping as used in the transformation method.
To clarify this for the reader, we have made the following changes throughout the manuscript:

- We have replaced "morphing-invariant" with "mapping-invariant" in the title and throughout the text. This emphasizes that the mechanical performance is invariant to the geometric mapping and microstructure reconfiguration of the lattice rather than to a continuous morphing process.
- In the Results section, we now explicitly describe the process of changing the material shape as microstructure and geometric reconfiguration to distinguish it from the elastic deformation that occurs under loading.
- Figure Updates: In Fig. 2 and Fig. 4, we have updated the labels to "Initial Configuration" and "Mapped Configuration" to reflect that these are distinct, programmed states rather than steps in a continuous deformation path.

These updates ensure that the manuscript accurately reflects the kinematic nature of the system, where performance is decoupled from the reconfigured state of the material.

2.4 General comment: authors consider in the system two kinds of springs: (real) springs (Fig. 1a) and effective lever springs (Fig. 1b). In the text both are interchangeably referred to as springs. It would help readability removing this ambiguity by introducing a fixed naming convention for each of the cathegories of the springs and strictly sticking to it throughout the text. This could be, for instance, springs and lever springs.

Reply: We thank the reviewer for this constructive suggestion to improve the readability of our manuscript. We agree that the interchangeable use of "springs" for both virtual model components and physical mechanisms could be confusing.
As suggested, we have introduced a fixed naming convention and applied it strictly throughout the revised main text and Supplementary Information:
1. The virtual model components used in theoretical lattice constructions (e.g., in Fig. 1 of the revised manuscript) are referred to as "linear springs" and "Willis springs".
2. Their physical realizations, which utilize rigid bar mechanisms as detailed in Figs. 2a and 3a of the revised manuscaript, are explicitly termed "linkage-based linear springs" and "linkage-based Willis springs", respectively.
We believe the term "linkage-based" accurately reflects the kinematic nature of the design and this distinction is now consistently applied throughout the text and figure captions.

2.5 General comment: the authors call the mechanism Willis coupling. In my understanding, it is simply linkage with another spring, fixed to the external world, hence displacements couple with forces, unlike, e.g., momentum density and strain, two distinct quantities. Is Willis coupling really substantiated here? Deeper clarification would be beneficial.

Reply: We thank the reviewer for this insightful comment regarding the substantiation of Willis coupling in our static system. We appreciate the opportunity to clarify the physical and theoretical correspondence between our grounded metamaterial and the classical dynamic Willis theory. We provide the following points to justify the use of this terminology:

- In the classical dynamic regime, Willis coupling originates from homogenization and denotes the cross-coupling between "monopole" and "dipole" quantities, i.e. between stress (dipole) and velocity (monopole), as well as between momentum (monopole) and strain (dipole) as general version of linear Cauchy elasticity. In the static limit ($\omega \to 0$), these relations undergo a mapping where velocity is replaced by displacement relative to the ground and momentum is replaced by grounded reaction forces (body forces). Thus, static Willis coupling manifests as the cross-coupling between stress and displacement, and between body force and strain, maintaining the fundamental 'monopole-dipole' interaction. The reason static Willis coupling has not been widely discussed is that most traditional homogenization is performed on free-standing materials. In a free-standing material, there are no external body forces in statics, which forces the Willis coupling terms to zero. While free-standing materials can exhibit Willis coupling in the dynamic regime. Here, we can consider the inertial force as the body force. Adding asymmetry in the microstructure would couple inertial force (or body force) with spatial gradients of velocity (or strain rate), described by taking time derivative to the moment equation in the Willis constitutive relation. Our grounded architecture provides the necessary external reference frame to host these body forces, thereby unlocking Willis coupling in the static limit.

- The substantiation of this term is further rooted in the transformation method developed in the manuscript to design GMMs. Transformation methods were previously utilized almost exclusively for wave control in optics and acoustics, where the form-invariance of governing equations is established for small-amplitude waves. However, our work unlocks a new application for the transformation method: Designing metamaterials with mapping-invariant and customized static displacement fields. Unlike wave-based transformation method, static transformation method for solid materials requires the material to be polar and exhibit the cross-coupling between stress and displacement, and between body force and strain under the coordinate transformation with nonuniform displacement gauges.

$$f_{ij}^d = [k_{ij}^d(u_j^d - u_i^d) \cdot n_{ij}^d - \hat{k}_{ij}^d u_i^d \cdot t_{ij}^d] n_{ij}^d, \tag{26}$$

$$\bar{f}_i^d = [\hat{k}_{ij}^d(u_j^d - u_i^d) \cdot n_{ij}^d - \tilde{k}_{ij}^d u_i^d \cdot t_{ij}^d] t_{ij}^d. \tag{27}$$

  where $\hat{k}_{ij}^d$ describe the cross-coupling and is the static Willis coupling coefficient as we introduce and define above. Thus, static Willis coupling is the requirement to achieve the mapping-invariant and customized displacement fields.

- Finally, we want to highlight that not every linkage connected to the external world can realize the specific Willis coupling required by transformation elasticity. The linkage must be carefully designed to satisfy the requirement that $(\hat{k}_{ij}^d)^2 = k_{ij}^d \tilde{k}_{ij}^d$ (according to the definitions of these three coefficients), meaning the stiffness matrix of the linkage-based Willis spring must be degenerate with a rank equal to 1. To meet this stringent requirement, we designed the four-bar linkage that comprises a torsional spring. Based on the equilibrium conditions between the torques on this torsional spring, the constitutive equations of the four-bar linkage read

$$\mathbf{f}_{AC}^d = \left[\frac{G^d \left(\mathbf{u}_A^d - \mathbf{u}_C^d \right) \cdot \mathbf{n}_{ij}^d}{L_{BC}^2} - \frac{G^d \mathbf{u}_C^d \cdot \mathbf{t}_{ij}^d}{L_{BC} L_{CD}} \right] \mathbf{n}_{ij}^d, \tag{28}$$

$$\mathbf{f}_E^d = \left[\frac{G^d \left(\mathbf{u}_A^d - \mathbf{u}_C^d \right) \cdot \mathbf{n}_{ij}^d}{L_{BC} L_{CD}} - \frac{G^d \mathbf{u}_C^d \cdot \mathbf{t}_{ij}^d}{L_{CD}^2} \right] \mathbf{t}_{ij}^d, \tag{29}$$

  where $\mathbf{u}_A^d$ and $\mathbf{u}_C^d$ are the displacements at nodes A and C, and G^d represents the effective torsional stiffness of the elastic cylinder. Comparing these to the theoretical Willis relations, we find that the Willis parameters are satisfied automatically by enforcing: $\frac{L_{CD}}{L_{BC}} = \frac{|\mathbf{B}_j \mathbf{n}_{ij}|}{|(\mathbf{B}_i - \mathbf{B}_j)\mathbf{n}_{ij}|}$ and $\frac{G^d}{L_{BC}^2} = K_{ij}^d$. This automatic satisfaction via geometric ratios is a key technical contribution, as it ensures the physical hardware exactly mimics the complex cross-coupling required by the transformation method. This degeneracy is the special physical signature that substantiates the mechanism as a true carefully designed static Willis spring.

We have revised the main text and SI to include this detailed theoretical context, ensuring the substantiation of Willis coupling in the static limit is clear to the reader.

2.6 Page 3: "Clearly, when the distance between nodes A and C changes...". BA can also rotate around B, thus changing AC distance without any torque. Please amend.

Reply: We thank the reviewer for pointing out this kinematic detail. The sentence in Page 4 (related to Fig. 3) has been amended to: "Clearly, when the distance between nodes A and C changes along the internal spring direction $\mathbf{n}_{ij}^d$ (where the node C is fixed), the bar BC rotates, producing torques on bars BC and CD."

2.7 Eq. (3): deserves reference to supporting material where expressions are explained in more detail.

Reply: We thank the reviewer for this suggestion. We have revised the text following Eq. (3) to explicitly direct the reader to the Methods section, where the step-by-step kinematic derivation of the Willis spring is provided. Additionally, we now refer to Supplementary Sections S3 and S6, which detail the numerical simulations and experimental testing that validate these expressions. These additions ensure that the mechanical basis for the Willis coupling coefficients is thoroughly substantiated for the reader.

2.8 General methodology comment: Currently the explanation is devised bottom-up through deduction steps starting from a simple mechanism by generalization, and is not fully straightforward to follow (especially rotation constraints are not fully clear, cf. Comment 2 above). All intermediate turns might be clouding unnecessarily the main working mechanism. Consider adding more specific details to improve readability or going top-down, i.e., introducing the ultimate mechanism and deductively reveal its functionality.

Reply: We thank the reviewer for this excellent suggestion regarding the explanation methodology, which served as the primary guideline for the major restructuring of our manuscript. We agree that the original bottom-up approach, which built the theory through incremental generalizations of simple mechanisms, may have obscured the core physical logic of the grounded metamaterial. As suggested, we have substantially revised the Results section to follow a top-down approach. The new version begins with the ultimate objective and the core methodology, as well as the necessary physical mechanisms (suppressed rotation and Willis coupling) required to satisfy transformation requirements. We then deductively reveals their physical realizations and the associated numerical and experimental results. The Results section is now reorganized into four logical distinct parts:

- Design GMMs based on the transformation method (The theoretical framework): Instead of deriving the concept mixed with mechanism realizations that could produce intermediate turns, we now immediately present the complete theoretical framework. The revised Results section begins by explicitly stating the high-level performance goal: decoupling mechanical behavior from microstructure and geometric reconfigurations. We frame coordinate transformation as the starting tool to derive the required lattice designs, providing a clear roadmap for the technical steps that follow. We show that once a material is remapped, internal forces become non-concurrent, and the rotational degrees of freedom must be suppressed by an external ground to provide the reaction body torques needed to balance these forces. The flow then moves naturally from achieving mapping-invariance (where $\mathbf{u}_i = \mathbf{U}_i$) to achieving customized displacement control ($\mathbf{u}_i^d \neq \mathbf{u}_i$) through the introduction of a displacement gauge ($\mathbf{B}_i$). We have consolidated the derivation of Willis coupling to show it as the mathematical consequence of the displacement gauge. To visually support this top-down logic, we have completely redesigned Fig. 1. It now follows a clear three-step progression: (i) Reference state: The baseline free-standing lattice. (ii) Mapping-invariant state: Introducing reconfiguration and the rotation-suppressed masses. (iii) Customized displacement state: Implementing the displacement gauge to derive the Willis coupling.

- GMMs with mapping-invariant displacement fields (Validation I: Invariance): Based on the theory established in Section 1, we demonstrate the first physical realization using grounded reconfigurable masses and linkage-based linear springs. This step independently validates the mapping-invariance capability, proving that the displacement field remains unchanged across different lattice configurations (rectangle, parallelogram, random) as predicted by the theory. We have added two figures (Figs. 2a and 2b) into Fig. 2. Fig. 2a shows the realization of the reconfigurable masses and linear springs using grooved bars and two-bar linkages (from Fig. 1 of the original manuscript), respectively. Fig. 2b illustrates the GMM assembled using the two-bar linkages for experimental testing.

- GMMs with customized displacement fields (Validation II: Customization): We then independently validate the second core capability: customized displacement control. We detail the realization of the linkage-based Willis spring and experimentally demonstrate how a specific displacement gauge generate nonstandard behaviors like asymmetric tension without bending. We redesigned a new figure (Fig. 3) based on Figs. 1 and 3 of the original manuscript. The realization, working principles, and experimental testing results of the linkage-based Willis spring are now clearly illustrated. In addition, the customized displacement control functions of the GMM assembled using these linkage-based Willis springs are demonstrated with both numerical and experimental results.

- GMMs with customized mapping-invariant displacement fields (Validation III: Mapping-invariant customization): Finally, we combine the findings from Sections 2 and 3 and implement another displacement gauge to demonstrate that the GMM can simultaneously achieve customized deformation (e.g., tension-induced buckling) and maintain this behavior invariantly across drastic changes in the lattice configuration. The experimental results are shown in Fig. 4, which are in Fig. 3 of the original manuscript.

By removing the intermediate steps and starting with the general theory, we believe the logical flow is now strictly deductive and much more straightforward to follow.

2.9 General comment: authors refer to restrained rotation of rigid masses. Those are, in my understanding, restrained due to two possible mechanisms: by the elastic lever mechanisms representing the springs (Fig. 1d), and by pinning/grounding them through magnets (Fig. 1e). If this is correct, similarly to Comment 4 above, consider specific naming convention for the two types of rotational restraints for better readability and using it consistently throughout the text.

Reply: We thank the reviewer for this insightful observation regarding the dual mechanisms that restrain the rotation of the rigid masses. We agree that distinguishing between the elastic forces provided by the linkage-based springs and the stiff kinematic constraints provided by the grounded supports is essential for mathematical and physical clarity. As suggested, we have introduced a specific naming convention to distinguish these two types of rotational restraints and have applied it consistently throughout the revised manuscript and figures:
1. Kinematic rotation constraint (or grounded kinematic constraint) refers to the constraint structure of two universal joints and a rigid bar connected to the ground via permanent magnets. Its primary purpose is to suppress the rotational degrees of freedom of the reconfigurable mass in a stiff manner, ensuring it functions as a point mass with only translational degrees of freedom as required by the transformation theory.
2. Elastic linkage-based restraint refers to the two-bar or four-bar linkage-based springs that provide the elastic potential energy and internal/grounded forces of the lattice. While these linkages are attached to the masses, their role is to define the material's stiffness and Willis coupling rather than to serve as the primary rotational anchor.
We have updated the labels in the figures and figure captions to clearly distinguish the kinematic rotation constraint (the universal joint assembly) from the elastic linkage-based restraint. We have added a clarification that the rotation is suppressed in a stiff manner by the kinematic constraint to match the theoretical assumptions, whereas the linkage-based springs provide the elastic response.

We have audited the text to ensure that when we refer to suppressed rotation, we are specifically referring to the kinematic grounding, and when we refer to restoring forces, we are referring to the linkage-based springs.

2.10 Fig. 2h: consider enlarging inset of point mass numbering.

Reply: We thank the reviewer for this detailed suggestion regarding readability. We have adopted this suggestion in the revised manuscript. Point mass numbering in the inset figure of Fig. 2h (now Fig. 2j) has been significantly enlarged with clear readability.

2.11 Figs. 2g-2h and elsewhere: horizontal axis' caption could be renamed (e.g., rigid mass ID/number). In my impression improving naming consistency throughout the entire text would improve readability significantly (points vs. masses, two kinds of springs, two kinds of rotation restraints, morphing/reconfiguration, all combined together was at times difficult for me to follow the main developments and it was not clear what or which mechanism authors exactly mean).

Reply: We thank the reviewer for this constructive feedback. We agree that the interplay of multiple physical mechanisms—grounding, rotation suppression, and Willis coupling—requires a high degree of terminological consistency to ensure the manuscript remains accessible. As suggested, we have performed a global audit of the manuscript and figures to standardize our naming conventions. Specifically, we have addressed the reviewer's points as follows:

1. Following the reviewer's suggestion, we have updated the horizontal axis labels in Figs. 2g, 2h, 3i and 3j (now in Figs. 2i, 2j, 4m, and 4n, and similar plots in the Supplementary Information) from "Number of point" to "Rigid mass number". This change accurately reflects that the data points represent the discrete translational response of the physical masses rather than arbitrary mathematical points.

2. To remove the ambiguity identified by the reviewer, we have established and strictly adhered to the following naming hierarchy throughout the revised text and figures: i. We now consistently use "rigid mass" or "reconfigurable mass" to describe the physical starburst structures. The term "point" is now reserved exclusively for specific geometric locations (e.g., "point A" on a linkage) or mathematical coordinates. ii. As previously detailed, "spring" refers to the components of the virtual lattice model, while "linkage-based spring" refers to the physical two-bar or four-bar linkage realizations. iii. We have clarified the distinction between the grounded universal joint assembly (the "kinematic rotation constraint", which suppresses rotation in a stiff manner) and the linkages (the "elastic linkage-based restraint", which provides the restoring elastic forces). iv. We have replaced "morphing" with "reconfiguration" or "mapping" when referring to the structural changes made to the lattice, reserving "deformation" for the elastic response of the material under external loads.

We believe these updates significantly improve the readability and consistency of the manuscript.

2.12 Page 5: effective properties of mechanical metamterials are often discussed in the literature. A discussion on, e.g., effective stiffness/anisotropy of GMMs as a function of their configuration would be interesting addition.

Reply: We appreciate the reviewer's comment on the effective mechanical properties of our grounded metamaterials with different configurations. We first revisit the continuum transformation elasticity and then perform homogenization for the grounded metamaterials derived from discrete transformation with different geometric mappings. Finally, we compare the effective elastic properties with those from continuum transformation. These discussions have been added into SI.
To formulate the continuum transformation, we first consider a virtual elastic solid occupied in $\mathbf{X}$ and assume that the virtual solid obeys Hooke's law. The virtual solid deforms with the displacement field $\mathbf{U(X)}$ in response to a given set of external loads or constraints. Our goal is to calculate the material properties of the transformed solid to realize the desired displacement field $\mathbf{u}(\mathbf{x})$ in a transformed coordinate $\mathbf{x}$. To facilitate the coordinate transformation, it is useful to write the elastic energy density of the virtual solid as

$$\mathcal{E} = \frac{1}{2} C_{ijkl} \frac{\partial U_j}{\partial X_i} \frac{\partial U_l}{\partial X_k}, \tag{30}$$

where C_{ijkl} denotes the elasticity tensor of the virtual solid, and the stress tensor reads $\Sigma_{ij} = C_{ijkl} \frac{\partial U_l}{\partial X_k}$. Note that we only consider linear deformations and Cauchy elasticity. To achieve the desired displacement field $\mathbf{u}(\mathbf{x})$ in the physical solid, we introduce a displacement gauge $\mathbf{B(X)}$ defined as [1]

$$\mathbf{u}(\mathbf{x}) = \left(\mathbf{B(X)}^T\right)^{-1} \mathbf{U(X)}, \tag{31}$$

where $\mathbf{B(X)}$ is an invertible matrix. The spatial derivative of the virtual displacement field then read

$$\frac{\partial U_j}{\partial x_i} = B_{pj} \frac{\partial u_p}{\partial x_i} + \frac{\partial X_q}{\partial x_i} \frac{\partial B_{pj}}{\partial X_q} u_p. \tag{32}$$

Implementing coordinate transformation by substituting Eq. (32) into Eq. (30), we obtain the elastic energy density in $\mathbf{x}$ as

$$e = \frac{1}{2} \left(\frac{\partial u_j}{\partial x_i} \sigma_{ij} + u_k f_k \right), \tag{33}$$

$$\sigma_{ij} = c_{ijkl} \frac{\partial u_l}{\partial x_k} + s_{ijk} u_k, \tag{34}$$

$$f_k = s_{ijk} \frac{\partial u_j}{\partial x_i} + d_{kl} u_l, \tag{35}$$

where $c_{ijkl} = \frac{1}{J} C_{pqst} \frac{\partial x_i}{\partial X_p} B_{jq} \frac{\partial x_k}{\partial X_s} B_{lt}$, $s_{ijk} = \frac{1}{J} C_{pqst} \frac{\partial x_i}{\partial X_p} B_{jq} \frac{\partial B_{kt}}{\partial X_s}$, $d_{kl} = \frac{1}{J} C_{pqst} \frac{\partial B_{kq}}{\partial X_p} \frac{\partial B_{lt}}{\partial X_s}$ with $J = \det(\frac{\partial x_i}{\partial X_j})$, and e, σ_{ij}, and f_k denote the elastic energy density, stress, and body force of the transformed solid, respectively. Clearly, the elastic energy density of the transformed solid e no longer

keeps its original form after the transformation. Instead, it has two parts: one comes from the strain energy density; the other arises due to the work done by the body force, a new term caused by the transformation. Further, the stress-strain relationship of the transformed solid is no longer Hooke's law, as noted by inspection of Eq. (34) and the definition of s_{ijk}, which depends on the transformation. Equation (34) shows that stresses in the transformed solid depend on both the displacement gradients and the displacement itself through a elastostatic Willis coupling tensor s_{ijk}. In a similar manner, body forces are determined by not only the displacement but the displacement gradients through the same Willis coupling tensor s_{ijk}. Secondly, the elasticity tensor of the transformed solid loses minor symmetry, e.g. $c_{ijkl} \neq c_{ijlk} \neq c_{jilk}$, leading to asymmetric stresses, a response characterizing elastic polarity. Once the two properties are satisfied in a solid material, the desired displacement behavior will be guaranteed. The observations here are identical with that derived from the discrete transformation shown in the main text.

Next, we homogenize the GMM design in the main text and calculate its effective elasticity tensors. The potential energy per unit area is calculated under a macroscopic displacement gradient $\mathbf{e}$ and a uniform displacement $\mathbf{u}$ over a unit cell of the GMM. For the triangular lattice demonstrated in the examples, we decompose the potential energy density $\bar{\varepsilon}$ into three parts caused by the three springs $\{i,j\}$, $\{i,k\}$, and $\{j,k\}$ in the unit cell. The potential energy density then reads

$$\bar{\varepsilon} = \bar{\varepsilon}_{ij} + \bar{\varepsilon}_{ik} + \bar{\varepsilon}_{jk}, \tag{36}$$

where

$$\bar{\varepsilon}_{ij} = \frac{k_{ij}^{d}}{2A}\left(\mathbf{e}\mathbf{r}_{ij}\mathbf{n}_{ij}^{d}\right)^{2} + \frac{\hat{k}_{ij}^{d}}{A}\left(\mathbf{e}\mathbf{r}_{ij}\mathbf{n}_{ij}^{d}\right)\left(\mathbf{u}\mathbf{t}_{ij}^{d}\right) + \frac{\tilde{k}_{ij}^{d}}{2A}\left(\mathbf{u}\mathbf{t}_{ij}^{d}\right)^{2}, \tag{37}$$

$$\mathbf{r}_{ij} = \mathbf{x}_i - \mathbf{x}_j, \tag{38}$$

and A is the area of the unit cell. The effective elasticity tensors of the GMM can be derived by

$$\bar{c}_{ijkl} = \frac{\partial^2 \bar{\varepsilon}}{\partial e_{ij}\partial e_{kl}}, \tag{39}$$

$$\bar{s}_{ijk} = \frac{\partial^2 \bar{\varepsilon}}{\partial e_{ij}\partial u_{k}}, \tag{40}$$

$$\bar{d}_{ij} = \frac{\partial^2 \bar{\varepsilon}}{\partial u_{i}\partial u_{j}}. \tag{41}$$

Calculating Eqs. (39-41) at the continuum limit, the effective elasticity tensors are equal to those derived from continuum transformation, $\bar{c}_{ijkl} = c_{ijkl}$, $\bar{s}_{ijk} = s_{ijk}$, and $\bar{d}_{ij} = d_{ij}$.

Next, we validate these relationships through two illustrative examples by calculating effective elastic properties using continuum transformation elasticity and performing homogenization for the grounded metamaterials. We assume a free-standing triangular lattice in the virtual space (as shown in the main text) with uniform spring constant K_0. Its effective elasticity tensor reads

$$\mathbf{C} = \begin{bmatrix} \lambda + 2\mu & \lambda & 0 & 0 \\ \lambda & \lambda + 2\mu & 0 & 0 \\ 0 & 0 & \mu & \mu \\ 0 & 0 & \mu & \mu \end{bmatrix}, \tag{42}$$

where $\lambda = \mu = \frac{\sqrt{3}K_0}{4}$.
Example I:
We select the displacement gauge $\mathbf{B} = \begin{bmatrix} 1 & -b(X_1) \\ 0 & 1 \end{bmatrix}$ and $\mathbf{x} = \mathbf{X}$ corresponding to the example demonstrated in Fig. 4a. Implementing this displacement gauge and coordinate mapping into Eqs. (33-35), transformed elasticity tensors derived from continuum transformation can be written as

$$\mathbf{c} = \mu \begin{bmatrix} 3+b^2 & 1 & -b & -2b \\ 1 & 3 & 0 & -3b \\ -b & 0 & 1 & 1 \\ -2b & -3b & 1 & 1+3b^2 \end{bmatrix}, \tag{43}$$

$$\mathbf{s} = \mu \begin{bmatrix} b\frac{\partial b}{\partial X_1} & 0 \\ 0 & 0 \\ -\frac{\partial b}{\partial X_1} & 0 \\ -\frac{\partial b}{\partial X_1} & 0 \end{bmatrix}, \tag{44}$$

$$\mathbf{d} = \mu \begin{bmatrix} (\frac{\partial b}{\partial X_1})^2 & 0 \\ 0 & 0 \end{bmatrix}. \tag{45}$$

Next, we perform homogenization for the grounded metamaterial shown in Fig. 4a based on Eqs. (36-41), the effective elasticity tensors are attained as

$$\bar{\mathbf{c}} = \frac{\sqrt{3}K_0}{4} \begin{bmatrix} 3+b_j^2 & 1 & -b_j & -2b_j \\ 1 & 3 & 0 & -3b_j \\ -b_j & 0 & 1 & 1 \\ -2b_j & -3b_j & 1 & 1+3b_j^2 \end{bmatrix}, \tag{46}$$

$$\bar{\mathbf{s}} = \frac{\sqrt{3}K_0}{4} \begin{bmatrix} \frac{2(b_j-b_k)}{a_0}b_j & 0 \\ 0 & 0 \\ -\frac{2(b_j-b_k)}{a_0} & 0 \\ -\frac{2(b_j-b_k)}{a_0} & 0 \end{bmatrix}, \tag{47}$$

$$\bar{\mathbf{d}} = \frac{\sqrt{3}K_0}{4} \begin{bmatrix} \frac{4(b_j-b_k)^2}{a_0^2} & 0 \\ 0 & 0 \end{bmatrix}, \tag{48}$$

where b_j denotes $b(\mathbf{X}_j)$. Comparing Eqs. (46-48) with Eqs. (43-45), it can be clearly seen that $\bar{\mathbf{c}} = \mathbf{c}$, $\bar{\mathbf{s}} = \mathbf{s}$, and $\bar{\mathbf{d}} = \mathbf{d}$ at the continuum limit.
Example II:
In the second example, we consider the parallelogram mapping corresponding to the grounded metamaterial in Fig. 4e, where $\nabla_X \mathbf{x} = \begin{bmatrix} 1 & 0 \\ \gamma & 1 \end{bmatrix}$, γ is a constant. Again, the transformed elasticity

tensors derived from continuum transformation can be written as

$$\mathbf{c} = \mu \begin{bmatrix} 3+b^2 & 1-\gamma b & -b & 3\gamma-2b+\gamma b^2 \\ 1-\gamma b & 3+\gamma^2 & \gamma & 2\gamma-3b-\gamma^2 b \\ -b & \gamma & 1 & 1-\gamma b \\ 3\gamma-2b+\gamma b^2 & 2\gamma-3b-\gamma^2 b & 1-\gamma b & 1+3\gamma^2-4\gamma b+3b^2+\gamma^2 b^2 \end{bmatrix}, \tag{49}$$

$$\mathbf{s} = \mu \begin{bmatrix} b\frac{\partial b}{\partial X_1} & 0 \\ -\gamma\frac{\partial b}{\partial X_1} & 0 \\ -\frac{\partial b}{\partial X_1} & 0 \\ (\gamma b-1)\frac{\partial b}{\partial X_1} & 0 \end{bmatrix}, \tag{50}$$

$$\mathbf{d} = \mu \begin{bmatrix} (\frac{\partial b}{\partial X_1})^2 & 0 \\ 0 & 0 \end{bmatrix}. \tag{51}$$

The homogenized elasticity tensors read

$$\bar{\mathbf{c}} = \frac{\sqrt{3}K_0}{4} \begin{bmatrix} 3+b_j^2 & 1-\gamma b_j & -b_j & 3\gamma-2b_j+\gamma b_j^2 \\ 1-\gamma b_j & 3+\gamma^2 & \gamma & 2\gamma-3b_j-\gamma^2 b_j \\ -b_j & \gamma & 1 & 1-\gamma b_j \\ 3\gamma-2b_j+\gamma b_j^2 & 2\gamma-3b_j-\gamma^2 b_j & 1-\gamma b_j & 1+3\gamma^2-4\gamma b_j+3b_j^2+\gamma^2 b_j^2 \end{bmatrix}, \tag{52}$$

$$\bar{\mathbf{s}} = \frac{\sqrt{3}K_0}{4} \begin{bmatrix} \frac{2(b_j-b_k)}{a_0}b_j & 0 \\ -\frac{2(b_j-b_k)}{a_0}\gamma & 0 \\ -\frac{2(b_j-b_k)}{a_0} & 0 \\ \frac{2(b_j-b_k)}{a_0}(\gamma b_j-1) & 0 \end{bmatrix}, \tag{53}$$

$$\bar{\mathbf{d}} = \frac{\sqrt{3}K_0}{4} \begin{bmatrix} \frac{4(b_j-b_k)^2}{a_0^2} & 0 \\ 0 & 0 \end{bmatrix}. \tag{54}$$

Comparing Eqs. (52-54) with Eqs. (49-51), the relationships $\bar{\mathbf{c}} = \mathbf{c}$, $\bar{\mathbf{s}} = \mathbf{s}$, and $\bar{\mathbf{d}} = \mathbf{d}$ still hold at the continuum limit.

2.13 Fig. 3f and related discussion in the text: it is not clear to me how the system is deformed. Are only the two points 3-4 displaced horizontally in the $-x_1$ direction? Additional explanation would be helpful.

Reply: We thank the reviewer for requesting clarification on this loading condition. The reviewer is correct: the deformation is induced by prescribing horizontal displacements (along the $-x_1$ direction) solely on the two central masses of the top and bottom boundaries, while the rest of the metamaterial remains free. This specific loading usually causes translation motion in free-standing materials, but triggers a zero-energy deformation mode in the GMM. We have refined the

corresponding description in the revised manuscript as "As shown in Figs. 4b and 4f, when the GMM is moved to the left by pushing the two central masses on the top and bottom boundaries...". In the revised figures, we have also replaced the abstract arrows with hand gestures (pushing or pulling icons) for an intuitive visual aid.

> **2.14** Discussion: "morphing-invariant" might be an overstatement in my opinion, cf. Comments 1 and 3 above.

Reply: We thank the reviewer for this remark. We agree that the term "morphing-invariant" could be misinterpreted to imply a continuous, elastic deformation between states, which does not accurately describe our discrete reconfiguration process. To ensure our terminology aligns precisely with the physical behavior of the system, we have made the following updates throughout the manuscript:
1. We have replaced "morphing-invariant" with "mapping-invariant" in the title and throughout the text. This term more accurately reflects that the mechanical performance is decoupled from the geometric mapping of the lattice architecture during reconfiguration.
2. As discussed in our responses to Comments 1 and 3, we now consistently use the term "reconfiguration" or "geometric mapping" when referring to the structural changes made to the lattice, reserving "deformation" exclusively for the elastic response to external loads.
We believe these changes address the reviewer's concern and provide a more accurate description of the physical principles demonstrated in this work.

> Supplementary material might benefit from the following changes:
> **2.15** Fig. S1: black font for C and D is poorly visible on the dark background.

Reply: Thanks for this useful remark. We have corrected the font for C and D to improve the visibility of labels in Fig. S1.

> **2.16** Page 2: "Universal joints and a rigid bar and is attached to the ground". See Comment 9 above; is the rotation restrained in a stiff manner, or is there elasticity in the rotational constraint?

Reply: We thank the reviewer for this question, which allows us to clarify the mechanical nature of the grounded supports. To address the reviewer's inquiry: the rotation of the rigid masses is restrained in a stiff manner. As we detailed in our response to Comment 9, we have introduced a clear distinction between the two mechanisms involving the masses:
1. The system comprising the two universal joints and the rigid bar is designed to be a stiff kinematic constraint. Its purpose is to physically suppress the rotational degrees of freedom of the reconfigurable mass while allowing free in-plane translation. This ensures the masses behave as point masses, which is a fundamental requirement of the transformation elasticity theory. In our prototype, the components (aluminum bars and universal joints) are chosen to ensure the rotational stiffness of this constraint is several orders of magnitude higher than the stiffness of the elastic linkages.

2. The elasticity of the metamaterial resides exclusively in the linkage-based springs (the linkages containing the elastic cylinders). These mechanisms provide the restoring forces and grounded reaction forces that define the material's elastic performance.
We have revised the text on Page 2 and in the Supplementary Information to explicitly state that the kinematic rotation constraint suppresses rotation in a stiff manner to satisfy the theoretical point-mass assumption. We have also clarified that the permanent magnet maintains this stiff grounded connection even as the material is reconfigured into different shapes.

2.17 S4: numerical simulations would benefit from more detailed information (mesh, type of simulation, solvers, etc.). This would certainly substantiate the space needed.

Reply: Thanks for the reviewer's comment regarding more details of numerical simulations. We have added those necessary details in Section S4 as followed:
Numerical simulations are performed using COMSOL Multiphysics for comparison. We first simulate the displacement-force responses of the Willis spring in the form of the four-bar linkage (see Figs. 3b and 3c). 3D Structural Mechanics Module is selected, and the four-bar linkage is modeled using the geometry from fabrication. The elastic cylinder is discretized using free tetrahedral elements, while the bars are meshed using swept meshes generated from free triangular elements on their surfaces. A mesh convergence study was performed, resulting in a final mesh containing approximately 3,000 elements and 80,000 degrees of freedom. Pin connections are simulated using Rigid Connector boundary conditions. To obtain the displacement-force responses, displacement boundary conditions are applied at nodes A and C individually, and reaction forces are calculated at nodes A and E. The linear system is solved using the MUMPS direct solver. In the simulations, Young's modulus and shear modulus of PLA are selected as 1.37 GPa and 0.51 GPa, respectively. The two values are measured from three-point bending and torsion tests.
The displacement fields of the grounded metamaterial are simulated using discrete models for simplicity. The grounded metamaterial is modeled under plane stress assumptions under the Solid Mechanics interface, where only reconfigurable masses are built as solid bodies. The reconfigurable masses are meshed using triangular elements with a maximum size of $0.1a_0$. The linear and Willis springs are modeled by point forces controlled by their constitutive equations (Eqs. 3 and 4), where Point Load interfaces with functions of displacements extracted from domain point probes are implemented. Rotational degree of freedom of the reconfigurable mass is suppressed by imposing global constraints on the displacements. To simulate rigid masses, Young's modulus of the masses is set sufficiently large. The equations are solved using the MUMPS direct solver. Numerical results for Figs. 2d - 2f and Figs. 3b - 3d are shown in Figs. S4 and S5, respectively, for comparison.

2.18 Figs. S6, S7, S9, S11, S12, S13, S14, S15: missing labels a., b., etc.

Reply: We thank the reviewer for this helpful correction. To improve the clarity of the Supplementary Information, we have made the following update: We have added the missing labels (a., b., etc.) to the individual panels in Figs. S4, S5, S6, S7, S9, S11, S12, S13, S14, and S15 (now Figs. S3, S4, S5, S8, S10, S11, S12, S13 and S14). These labels now correspond directly with the descriptions provided in the figure captions.

Clarity and context
The main message of the contribution is currently not fully clear to me, although morphing-invariant metamaterials is stands out. The issue I have might be more related to small inconsistencies and gaps in the precise methodology and design, which might improve with the suggestions listed above. The proposed concept relates well to work within mechanical metamaterials.
References
The body of existing literature is sampled well, with no particular suggestions from this reviewer.
Your expertise
Experimental validation is at the edge of the expertise of this reviewer.

Reply: We thank the reviewer for their valuable feedback and for noting that our concept relates well to existing work in mechanical metamaterials. We appreciate the reviewer's perspective that the main message could be further clarified. To address this, we have substantially revised the manuscript to resolve the small inconsistencies and gaps identified, focusing on the following areas:
1. We have revised the Introduction and Discussion to state the core contribution more explicitly: We have unlocked the transformation method for designing materials with mapping-invariant and customized static displacement fields. The main message is that the grounded aspect is a fundamental physical requirement to decouple mechanical performance from geometric reconfiguration, and Willis coupling allows for customized non-standard behaviors, such as asymmetric tension without bending, that are impossible in traditional free-standing systems.
2. As suggested in our response to Comment 8, we have completely restructured the Results section to follow a top-down approach. We now start with the high-level objective and methodology of decoupling behavior from reconfiguration and deductively reveal why a grounded architecture, rotation suppression, and static Willis coupling are necessary physical requirements for customized mapping-invariant displacement fields. This systematic reorganization ensures that the methodology and design choices are presented as logical consequences of the transformation theory.
3. To address the gaps in clarity mentioned by the reviewer, we have performed a global audit of the manuscript and figures to standardize our terminology. As detailed in our previous responses, we have introduced fixed naming conventions: "Linear spring" (Theoretical) vs. "linkage-based spring" (Physical realization); "Kinematic rotation constraint" (Stiff grounding) vs. "Elastic linkage-based restraint" (Elastic linkage); "Geometric mapping/reconfiguration" vs. "Load-induced deformation"; "Rigid mass" instead of "point" for physical components.
4. We have completely redesigned all figures to mirror this improved logic and terminology. These figures now guide the reader through the transition from a reference lattice to a mapping-invariant grounded system, and finally to a customized Willis-coupled metamaterial.
We believe these systematic changes, prompted by the reviewer's insightful comments, have significantly enhanced the clarity and context of our work, ensuring the main message and technical contributions are now presented in a straightforward and consistent manner.

References

[1] F. Guevara Vasquez, G. W. Milton, D. Onofrei, P. Seppecher, Transformation elastodynamics and active exterior acoustic cloaking, in: Acoustic Metamaterials, Springer, 2013, pp. 289–318.
